# Anatomy and transcript profiling of gynoecium development in female sterile *Brassica napus* mediated by one alien chromosome from *Orychophragmus violaceus*

**DOI:** 10.1186/1471-2164-15-61

**Published:** 2014-01-23

**Authors:** Wen-qin Fu, Zhi-gang Zhao, Xian-hong Ge, Li Ding, Zai-yun Li

**Affiliations:** 1 National Key Lab of Crop Genetic Improvement, National Center of Crop Molecular Breeding Technology, National Center of Oil Crop Improvement (Wuhan), College of Plant Science and Technology, Huazhong Agricultural University, Wuhan 430070, P. R. China; 2 Key Lab of Qinghai Province for Plateau Crop Germplasm Innovation and Utilization, Academy of Agricultural and Forestry Sciences, Qinghai University, Xining 810016 Qinghai Province, P. R. China

**Keywords:** *Brassica napus*, Gynoecium, Ovule, Female sterility, Transcriptome

## Abstract

**Background:**

The gynoecium is one of the most complex organs of angiosperms specialized for seed production and dispersal, but only several genes important for ovule or embryo sac development were identified by using female sterile mutants. The female sterility in oilseed rape (*Brassica napus*) was before found to be related with one alien chromosome from another crucifer *Orychophragmus violaceus*. Herein, the developmental anatomy and comparative transcript profiling (RNA-seq) for the female sterility were performed to reveal the genes and possible metabolic pathways behind the formation of the damaged gynoecium.

**Results:**

The ovules in the female sterile *Brassica napus* with two copies of the alien chromosomes (S1) initiated only one short integument primordium which underwent no further development and the female gametophyte development was blocked after the tetrad stage but before megagametogenesis initiation. Using *Brassica*_ 95k_ unigene as the reference genome, a total of 28,065 and 27,653 unigenes were identified to be transcribed in S1 and donor *B. napus* (H3), respectively. Further comparison of the transcript abundance between S1 and H3 revealed that 4540 unigenes showed more than two fold expression differences. Gene ontology and pathway enrichment analysis of the Differentially Expressed Genes (DEGs) showed that a number of important genes and metabolism pathways were involved in the development of gynoecium, embryo sac, ovule, integuments as well as the interactions between pollen and pistil.

**Conclusions:**

DEGs for the ovule development were detected to function in the metabolism pathways regulating brassinosteroid (BR) biosynthesis, adaxial/abaxial axis specification, auxin transport and signaling. A model was proposed to show the possible roles and interactions of these pathways for the sterile gynoecium development. The results provided new information for the molecular mechanisms behind the gynoecium development at early stage in *B. napus*.

## Background

The gynoecium, located in the fourth and innermost whorl of a flower, is the female reproductive organ of flowering plants which has specialized functions for successful pollination, seed maturation and seed dispersal. Although it is a highly complex organ that differs widely in form between species, most gynoecia of different angiosperms have a set of common structures: an apical stigma, a style, and a basal ovary, which encloses the ovules [1,2]. In *Brassicaceae* family, including the model plant *Arabidopsis thaliana* and important *Brassica* crops, gynoecium is composed of two fused carpels and three common parts above. The stigma plays a key role in pollen binding and recognition and participates in the induction of pollen germination [3]. The style connects the stigma with the ovary and harbors the transmitting tract essential for pollen tube growth. The ovary contains the ovules that develop into seeds after fertilization [4,5]. The ovule contains the funiculus, the chalaza which forms outer and inner integuments, and the nucellus which is covered by the integuments and in which the embryo sac representing the megagametophyte forms [6,7]. Incomplete or abnormal development in any part of gynoecium can cause female sterility or reduced fertility, which has been observed in various plants, including *Arabidopsis* and *Brassica* crops [8,9]. The female sterile mutants provide the suitable materials for elucidating the genetic control of the gynoecium development.

As the gynoecium is one of the most complex and important organs of flowering plants, increasing researches focused on the genetic control of its development by using female gametophytic mutants, especially from *Arabidopsis*. Several genes important for ovule integument or embryo sac development have been identified, such as *SIN1*, *BEL1*, *INO*, *ATS*, *ANT*, *TSO1*, *HLL*, *NZZ*, *SIN2*, *WUS, PFS2*[7,10], *DYAD*[11] and *VDD*[12]. In addition, it was proposed that adaxial–abaxial polarity mechanisms were required for integument formation [13,14]. The auxin concentration gradient was found to determine cell fates in the embryo sac [15]. Two genes (*AGO5* and *AGO9*) were shown to control female gamete formation and megagametogenesis by two independent small RNA pathways [16,17]. Brassinolide was suggested to plays a previously unrecognized role in the development of gynoecia and outer integument of the ovule [18]. Some other genes or gene families like *STY*, *SHI*, *HECATE*, *SHATTERPROOF*, *JAIBA*, *CLV1* and *SPT* were identified for gynoecium development, including stigma, style, septum, transmitting tract and carpel margin tissues [1,2,5,19-22]. Recently, by applying whole genome microarray and Next Generation Sequencing (NGS) techniques, hundreds of genes were found to be specific for female gametophyte genes by comparative expression profiling between wild plants and mutants [23-25].

The female sterile mutants from spontaneous or artificial mutations were rarely reported in the important oilseed rape *Brassica napus* L. [8]. In our pervious study, complete female sterility was observed in one *Brassica napus- Orychophragmus violaceus* addition line which contained all 38 chromosomes from *B. napus* and one or two copies of one particular chromosome from *O. violaceus*[26]. It seemed that its pistils stopped to develop at early stage. So we inferred that certain genes related to early process of pistil formation on this alien chromosome silence the homologous genes in *B. napus*, or these alien genes interfere the normal pistil development of *B. napus*. In this study, we compared the developmental anatomy and transcript profiling using RNA-Seq technique of the gynoecia between the female sterile line and donor *B. napus*. A number of candidate genes and related pathways were revealed, which provided new insights into the genetic and biochemical controls for early stage of pistil development in *B. napus*. In addition, the sequence datasets serve as a valuable resource for novel gene discovery in *O. violaceus*.

## Results

### Developmental anatomy of female sterility and donor *B. napus*

**Figure 1:**
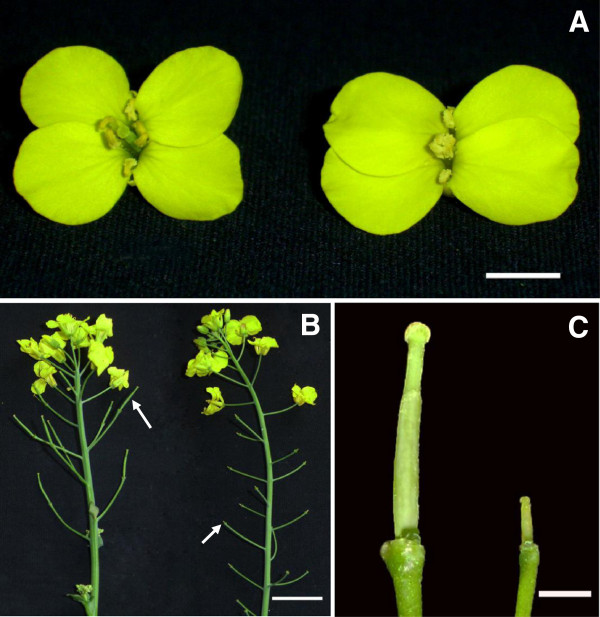
**Morphology of flower, gynoecium and seed setting in H3 and S1. A**, Open flower of H3 (left) and S1 (right), the gynoecium was short and hidden in the stamens in S1, bar = 5 mm. **B**, Inflorescence of H3 (left) and S1 (right), the arrows indicate the siliques, bar = 20 mm. **C**, Gynoecium at open flower stage of H3 (left) and S1 (right), the sepals, petals and stamens were removed, bar = 2 mm.

**Figure 2:**
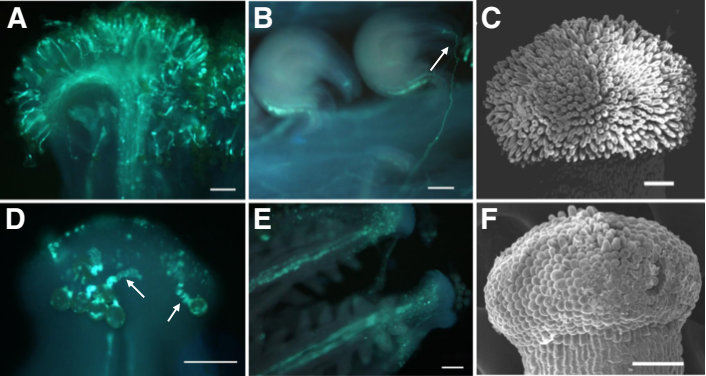
**Pollen germination, pollen tube elongation and papilla cells in H3 (A, B and C) and S1 (D, E and F).** Pollen grains germinate on stigma of H3 **(A)** and S1 **(D)** at 6 h after hand-pollinations, the pollen tubes are screwy (arrows in **D**) and fail to penetrate into papilla cells in S1. At 72 h after self-pollination, the pollen tubes have passed into the micropyle (arrow in **B**) of ovule of H3 **(B)**, but no pollen tubes are found in S1 ovary **(E)**. Papilla cells of S1 **(F)** are much shorter than that of H3 **(C)**. **A-B**, **D-E**, Fluorescence micrographs. **C** and **F**, Scanning electron micrographs. Bar = 100 μm.

The female sterile disomic addition line (S1) with all 38 chromosomes from *B. napus* and two copies of the chromosome from *O. violaceus* produced the progenies with similar phenotype, except the different female fertilities, after it pollinated donor *B. napus* (H3). The female sterile plants carried one or two copies of the *O. violaceus* chromosome (2n = 39, 40), while female fertile plants had the same chromosome number as normal *B. napus* (2n = 38). The female sterile plants were indistinguishable from fertile ones during vegetative growth period, for they only failed to produce normal gynoecia (Figure 1). They showed completely female sterility, and produced no seeds after self-fertilization or pollinated by *B. napus*. But they developed normal petals and anthers with abundant stainable pollen grains (90-95% stainability), for H3 showed normal seed setting when pollinated by the female sterile plants. In comparison with H3, the gynoecia of S1 were slow-growing and much shorter from early development stage (flower buds ~2 mm) to flowering (Figure 1A, C), and the pistils showed no indication of swelling from fertilization and fell off after the flowers opened some time (Figure 1B). Correspondingly, the flowering duration of S1 plants was prolonged till seed maturity of H3, because they had no seed setting and produced more flowers. Pollination analysis found that few pollen grains of itself or H3 could adhere and germinate on S1 stigmas 6 h after pollination (Figure 2A, D). However, although pollen tubes could grow spirally over the surface of papillae cells (Figure 2D), they hardly penetrated into stigmas or styles and then failed to reach abnormal ovules even 48 or 72 h after pollination (Figure 2E). Further observation showed that papilla cells on S1 stigmas were short, small and not fingerlike as those of H3 at maturation stage (Figure 2C, F).

**Figure 3:**
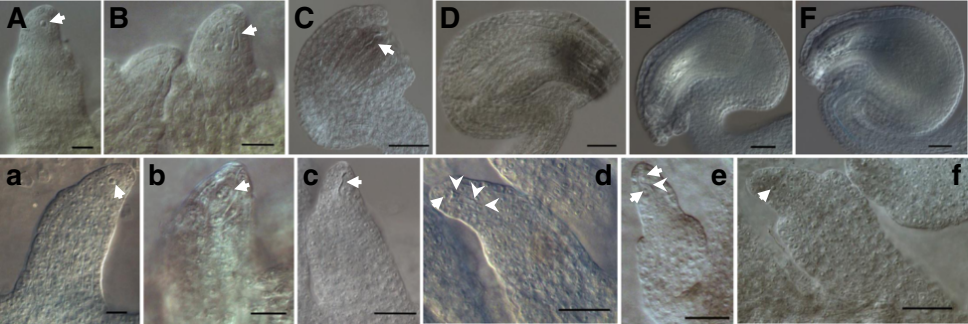
**Stages of ovule development in H3 (A-F) and S1 (a-f).** Photomicrographs of chloral hydrate cleared ovules. **(A, a)** MMC has differentiated (arrow) and inner integument has initiated. **(B, b)** Megaspore tetrad has been produced (arrow) but outer integument primordia form only in H3. **(C, c)** Postmeiotic ovules. The arrows indicate degenerated haploid spores in S1. In H3 the integuments extend to nucellus, whereas, integument arrests at initiative stage in S1. **(D, E, F)** Mononuclear, two-nuclear and mature embryo sacs of H3, respectively. **(d, e, f)** S1 ovules at the same stage as normal mature embryo sac but abnormal nucellus without integument surrounding: one nucleus (arrows) and three degenerated spores (arrowheads) in **d**, two nuclei (arrows) and a ray of residuum (arrowheads) in **e**, one small nucleus likely without differentiation (arrow) in **f**. MMC: Megaspore Mother Cell. Bar = 20 μm in **A-B**, **a-b** and 50 μm in **C-F**, **c-f**.

The developmental processes of the gynoecia in S1 and H3 were comparatively observed from the cleared ovules to determine the stage and tissue for the occurrence of the female sterility. In H3, the gynoecia formation was characterized by the normal development of the ovule and embryo sac (Figure 3A-F). In S1, ovule primordia appeared normally from the placenta in which one megaspore mother cell (MMC) was included (Figure 3a), and four daughter cells were produced from the meiotic division of one MCC in the ovule (Figure 3b). But the integuments could not initiate or arrest at the initiative stage (Figure 3a-c), and only a small protuberance at the chalaza region was left (Figure 3d-f). Then in incomplete embryo sacs, the four meiotic products showed several patterns of degradation: all degraded (Figure 3c), one nucleus and three degenerated arranged in a line (Figure 3d), a ray of residuum and two nuclei surrounded (Figure 3e), and only one residue left in the nucellus (Figure 3f). The location of residual cells at the micropylar end (Figure 3d) but the functioning megaspore at the chalazal end suggested the change of the normal polarity of cell location in embryo sac. These anatomical investigations revealed that the embryo sac development might stop after the meiotic division of the MMCs.

### Illumina sequencing and mapping of transcripts

According to the morphology of different-stage gynoecia of S1 and H3 observed above, the development of their gynoecia in the flower buds < 2 mm matched each other, but deviation started in the flower buds ~2 mm with gynoecia ~1.2 mm, and expanded with the successive growth of flower buds (Additional file 1: Figure S1). So the gynoecia from 1.5-3 mm flower buds from S1 and H3 were collected for analysis of changes in gene expressions responsible for their different developmental patterns. From mRNA sequencing, 7,568,113 and 7,059,416 raw reads were obtained from S1 and H3, respectively, and 7,502,922 (99.14%) and 6,990,973 (99.03%) clean reads were left, after “dirty” raw reads were removed. To identify genes corresponding to reads in each library, the clean reads were aligned to the *Brassica*_95k_unigene reference genome using the Short Oligonucleotide Alignment Program 2 (SOAP2) aligner, allowing for two bases mismatch. Of the clean reads, more than 62% matched either to a unique or multiple genomic positions, and 4,106,692 and 3,922,411 uniquely matched reads were used for gene expression analysis of each library. The detailed alignment statistics was displayed in Table 1.

**Table 1:** Summary of alignment statistics of RNA-Seq in H3 and S1

	**H3**		**S1**	
**Total reads**	6990973		7502922	
**Total mapped reads**	4396449	62.89%	4652862	62.01%
**Perfect match**	2440840	34.91%	2564012	34.17%
**< = 2 bp mismatch**	1955609	27.97%	2088850	27.84%
**unique match**	3922411	56.11%	4106692	54.73%
**Mapped genes**	27653		28065	

### Screening of differentially expressed genes (DEGs)

**Figure 4:**
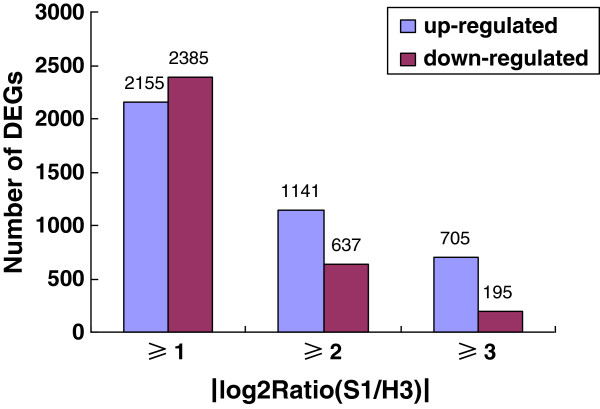
**Comparison of expression of the mapped genes between S1 and H3.** Columns denote the number of DEGs with an expression ratio within the stated range.

After mapped to reference genome, a total of 28,065 and 27,653 unigenes were identified and transcribed in S1 and H3, respectively, suggesting that newly initiated transcription occurred in S1. The 26610 unigenes were overlapped between these two transcript libraries, 1455 expressed only in S1 and 1043 only in H3. Using false discovery rate (FDR) ≤ 0.001 and the absolute value of |log2Ratio| ≥ 1 as criteria, 4540 unigenes exhibited different expressions between S1 and H3, including both up-regulated and down-regulated genes in S1. Of the 2155 up-regulated unigenes, 192 genes were uniquely expressed in S1, and 37 of the 2385 down-regulated unigenes expressed only in H3. Moreover, 1141 unigenes were up-regulated and 637 down-regulated with |log2Ratio| ≥ 2, 705 and 195 were up- and down-regulated with |log2Ratio| ≥ 3, respectively (Figure 4).

### Gene Ontology analysis and pathway analysis of DEGs

**Figure 5:**
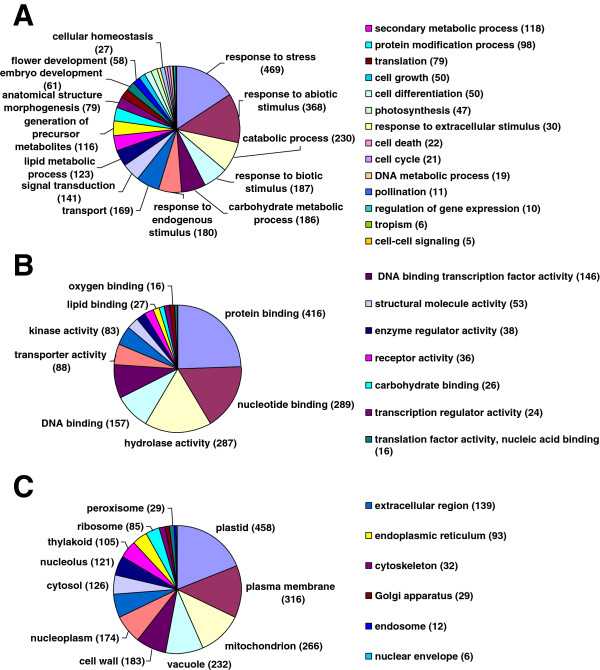
**Functional categorization of GO terms in DEGs with RPKM** ≥ **50 and fold change** ≥ **2 by blast2GO.** The genes are categorized based on Gene Ontology (GO) annotation and the proportion of each category is displayed based on: Biological process **(A)**; Molecular function **(B)**; Cellular component **(C)**.

To further clarify the functional differences between S1 and H3, DEGs with the criteria of RPKM ≥ 50 and fold change ≥ 2 were annotated according to function annotation convention. Gene ontology (GO) categories were assigned to significant DEGs based on the TAIR GO slim provided by blast2GO (GO enrichment analysis using hypergeometric test for 4540 unigenes was showed in Additional file 2: Table S1). A total of 1987 DEGs could be categorized into 60 categories (filtered by Seq: cutoff = 5.0) (Figure 5), involving metabolism, growth, development, catabolic and apoptosis. Based on the molecular functions, 15 categories genes involved in protein binding (416 genes, 24%), nucleotide binding (289 genes, 17%), hydrolase activity (187 genes, 17%), DNA binding (157 genes, 9%) and so on. Regarding biological process, the genes were finally classified into 28 categories, the most over-represented GO term was response to stress (469 genes), followed by response to abiotic stimulus (368 genes), catabolic process (230 genes), response to biotic stimulus (187 genes). There were several related processes, including flower development (58 genes), cell growth (50 genes), cell differentiation (50 genes), photosynthesis (47 genes), cell death (22 genes), cell cycle (21 genes) and pollination (11 genes). Among the cellular component categories, plastid (458 genes) and plasma membrane (316 genes) were the dominant groups, followed by mitochondrion (266 genes), vacuole (232 genes) and cell wall (183 genes).

Pathway analysis for the 4540 unigenes was performed using hypergeometric test. A total of 20 different metabolic pathways were found with Q ≤ 0.05 (Table 2). The most represented pathways were linoleic acid metabolism (17 genes), biosynthesis of secondary metabolites (389), metabolic pathways (681), glycolysis/gluconegenesis (63) and flavonoid biosynthesis (52).

**Table 2:** Pathway enrichment analysis of DEGs

**KEGG**^**a **^**pathway**	**DEGs with pathway annotation (2333)**	**All genes with pathway annotation (30493)**	**Qvalue**^**b**^
Linoleic acid metabolism	17 (0.73%)	46 (0.15%)	2.396666e-06
Biosynthesis of secondary metabolites	389 (16.67%)	3987 (13.08%)	6.888993e-06
Metabolic pathways	681 (29.19%)	7643 (25.06%)	4.918936e-05
Glycolysis/Gluconeogenesis	63 (2.7%)	446 (1.46%)	4.918936e-05
Flavonoid biosynthesis	52 (2.23%)	345 (1.13%)	4.918936e-05
alpha-Linolenic acid metabolism	38 (1.63%)	227 (0.74%)	7.964526e-05
Circadian rhythm - plant	48 (2.06%)	351 (1.15%)	1.109326e-03
Glucosinolate biosynthesis	22 (0.94%)	127 (0.42%)	3.626965e-03
Carbon fixation in photosynthetic organisms	45 (1.93%)	346 (1.13%)	4.489689e-03
Valine, leucine and isoleucine degradation	29 (1.24%)	194 (0.64%)	4.707493e-03
Ribosome biogenesis in eukaryotes	68 (2.91%)	590 (1.93%)	5.175354e-03
Arginine and proline metabolism	37 (1.59%)	295 (0.97%)	2.056500e-02
Protein processing in endoplasmic reticulum	91 (3.9%)	887 (2.91%)	2.378769e-02
Glutathione metabolism	37 (1.59%)	301 (0.99%)	2.491330e-02
Glycine, serine and threonine metabolism	28 (1.2%)	213 (0.7%)	2.784337e-02
Starch and sucrose metabolism	101 (4.33%)	1025 (3.36%)	3.905783e-02
Galactose metabolism	28 (1.2%)	224 (0.73%)	4.579364e-02
Zeatin biosynthesis	27 (1.16%)	214 (0.7%)	4.579364e-02
Propanoate metabolism	22 (0.94%)	165 (0.54%)	4.579364e-02
Tropane, piperidine and pyridine alkaloid biosynthesis	17 (0.73%)	117 (0.38%)	4.579364e-02

^a^KEGG = Kyoto Encyclopedia of Genes and Genomes.

^b^Pathways with Qvalue ≤ 0.05 are significantly enriched in DEGs.

### DEGs specific to S1 and H3 plants

As S1 carried the alien *O. violaceu* chromosomes, it was understandable that DEGs specific to S1 (192 unigenes) were more than those of H3 (37). Significantly enriched GO terms of the two sets of DEGs were listed in Additional file 3: Table S2 and Additional file 4: Table S3. For the 192 ones, there were 51 enriched GO terms including 22 mapped to biological process ontology, 3 mapped to molecular function ontology and 26 mapped to cellular component ontology. For the 37 ones, only 2 GO terms (carbohydrate metabolic process and metabolic process) belonging to biological process ontology were significantly enriched. No GO terms were specific to H3 plants for molecular function and cellular component.

### DEGs for steroid biosynthesis and metabolic process

Fifteen unigenes involved in steroid biosynthesis were differentially expressed between S1 and H3. For phytosterol, twelve of the fifteen unigenes covered brassinosteroid (BR) and stigmasterol biosynthesis, which were all down-regulated in S1 plants. These genes were identified to encode proteins LUP2 (JCVI_38125 and JCVI_42543), CAS1 (JCVI_33856 and JCVI_20625), SMO1 (JCVI_2267), C-14 Sterol Reductase (FACKEL) (JCVI_39052), SMO2 (JCVI_14504), DWARF5 (JCVI_10359 and JCVI_40245) and DWARF1 (JCVI_9676, JCVI_21095 and JCVI_7150). Down-regulation of these genes could affect the normal biosynthesis of brassinosteroid, which might have a role in gynoecium and ovule development [18]. Furthermore, one unigene (JCVI_27911) encoding a DON-Glucosyltransferase termed *UGT73C5* involved in BR metabolic process was observed to highly and only expressed in S1 plant (log2 Ratio(S1/H3) >19). In *Arabidopsis*, the *UGT73C5* was found to regulate BR activity by catalyzing the 23-O-glucosylation of BL and castasterone, and overexpression of *UGT73C5* resulted in BR-deficient phenotypes [27]. In S1, the BR activity was likely reduced by both biosynthesis and metabolic process, and then affected the S1 gynoecium and ovule development.

### DEGs involved in floral organ, embryo sac or ovule development

In the flower development GO terms, 35 unigenes showed different expression levels. Among 9 unigenes for floral organ or whorl development, 5 encoding ABCB19 (JCVI_15407 and JCVI_35748), SHP2 (JCVI_13562), HEC1 (JCVI_35218) and SPT (JCVI_28865) were involved in carpel development [20,28-30], 3 encoding SRS5 (JCVI_29726) and TAA1 (JCVI_9912 and JCVI_18521) in gynoecium development [19,31], only 1 in sepal development instead of carpel or gynoecium development. All 8 female organ-related genes were down-regulated. In the DEGs, some others for embryo sac or ovule development were observed. For example, the genes encoding VDD (JCVI_5595 and JCVI_25612) were found to be a direct target of the MADS domain ovule identity complex and affect embryo sac differentiation in *Arabidopsis*[12]; the gene encoding AGO5 (JCVI_17792) was a putative effector of small RNA (sRNA) silencing pathways, an insertion of which inhibited the initiation of megagametogenesis [17]; the gene which encoded a protein disulfide isomerase, PDIL2-1 (JCVI_23062), its truncation would function in sporophytic tissues to affect ovule structure and impede embryo sac development, thereby disrupting pollen tube guidance [32]. In addition, the genes which encoded SEP2 (JCVI_18581), PI (JCVI_17089), AP3 (JCVI_7877), EDA14 (JCVI_13115), EDA17 (JCVI_16034) were also involved in female gametophyte or ovule development [28,29,33]. Most of these genes were down-regulated except *AGO5*, *PI* and *EDA14*.

### Verification of DEGs by qRT-PCR

**Figure 6:**
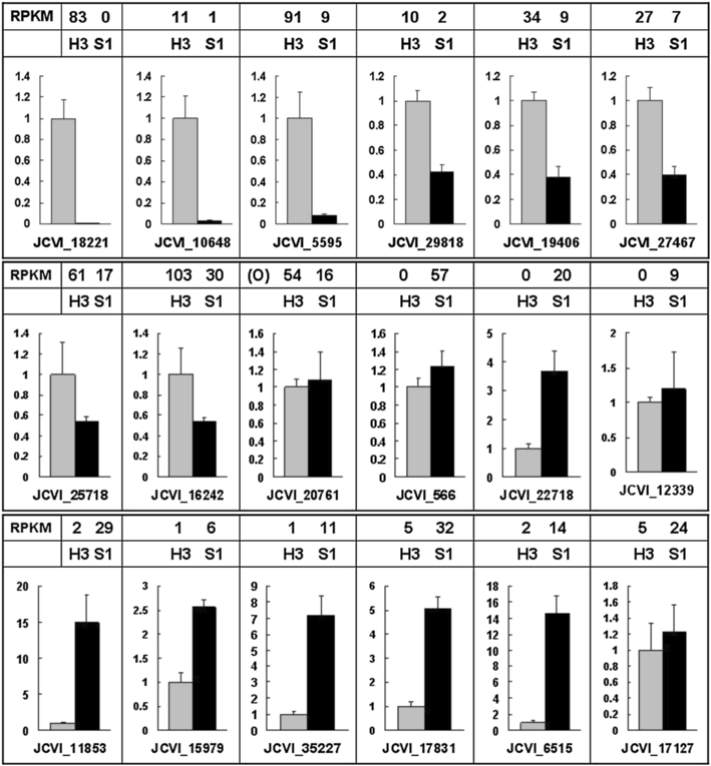
**RT-PCR confirmation of the differentially expressed genes between H3 (gray columns) and S1 (black columns).** Columns and bars represent the means and standard error (n = 3), respectively. The gene expression levels from RNA-Seq data are added on the top of each gene. RPKM: Reads Per Kb per Million reads, O: the change trend of gene expression is the opposite to the result of RNA-Seq.

To confirm that the unique-match genes from the Illumina sequencing and bioinformatics analysis were indeed differentially expressed, a total of 21 genes were selected randomly from the DEGs related to flower development (including gamete, embryo sac, integument, pollen or other floral organ development) for quantitative RT-PCR assays. With the gene-specific primers designed (Table 3), the relative transcript levels in S1 and H3 were compared with those of RNA-seq data (RPKM value). For 17 out of the 21 genes, qRT-PCR analysis revealed the same expression trends as the RNA-seq data, despite some quantitative differences. Among 18 of the 21 genes with different expression levels between S1 and H3 (Figure 6), 8 were repressed, 9 induced, and 1 opposed to RNA-Seq in S1, respectively. For example, JCVI_11853, a transcription factor which regulated flower development, was up-regulated 14.9 times in S1 than in H3 by qRT-PCR, nearly the same ratio as the one (29 to 2) from RNA-seq. Moreover, the six related genes, JCVI_18221 (*SLG*), JCVI_5595 (*VDD*), JCVI_19406 (*PHV*), JCVI_25718 (*CNA*), JCVI_16242 (*PHB*) and JCVI_22718 (*YAO*), showed the same expression trends revealed by qRT-PCR and RNA-seq, confirming the reliability of RNA-seq data.

**Table 3:** The corresponding primers of qRT-PCR

**Primer sequences**	**Amplified unigenes**
GGATGGAATAGCTGGAATCATT (F) TCATAACTGGATACCTGACTGTTGG (R)	JCVI_24628
CGTGAACGAGTCCAATTACCT (F) CGTACTCCAAGACAGGCCATA (R)	JCVI_36924
TCCTTCTCACATCAGCTCACAAGT (F) AACCAATGTAGTCTCAAGCATGTC (R)	JCVI_25718
TTCCGCTGAAGCTCTCACTCT (F) CTGCATGATCTTAGCGAACTCAG (R)	JCVI_16242
CAGTATCTGAAGGAGAACGGTAGC (F) AAGAACCGATGGAGTGAAGCTC(R)	JCVI_15979
CTACACAATCATAACAAGCCAATGC (F) TTGAAGATAGGAAGACTAGGACCAC (R)	JCVI_17831
ACGCAAGCCTCGTCCTCCTT(F) AAGCTCTGCCACATGAACCG (R)	JCVI_18221
AGCGACTGCCTCTAAGTGACG (F) TGCCACACGATGTTCTCTGG (R)	JCVI_10648
GGTGACAGTCCTGGATTCTTCA (F) TAAGCATCTCCGTACCATCTGG (R)	JCVI_5595
TTGTGGACGATAATCTGGTGAACAG (F) TCTTCGCTAGTAGCCTGAATCACAT (R)	JCVI_29818
CGTAGCAAGTGGTCAGCAAC (F) CCAGTCAACAGCAGTTCCTG (R)	JCVI_19406
TTGGACTATGGTAGCTGGAGGAG (F) CCATTGAAGGTAGCAGCAACATC (R)	JCVI_27467
GACCGTTACTCCATCCGCAT (F) TCTCGCAGAAGCACTCTCGT (R)	JCVI_20761
TAACATTGTGCTTAGTGGTGGAACC (F) GTCCAGATTCGTCATACTCAGCCTT (R)	JCVI_566
TAGTGACCTTGCTGCATCTGGAG (F) CCAACAGAATCCTTATGACAGCCT (R)	JCVI_22718
AACGAGACTTACCAGCTTCAGG (F) TGGAGAGAATGCTGATGCAG (R)	JCVI_12339
GGTGAGATCAACGAGGACAACGT (F) CTTCCACATGCGCCTCTTCTTC (R)	JCVI_18334
CACTTACGCCGACGAGCTTC (F) GAGTAACGGAACCGCCACAC (R)	JCVI_11853
GATCCTCTTCGTCTCTTCTTCAT (F) TTGGCACCATGTGATCGTAG (R)	JCVI_35227
CGAAGCCTTCTGTCTCATCG (F) ACACCGTCTCCGTCTCTGTC (R)	JCVI_6515
ATAATGGAGCCGTGGAGGTG (F) AATGGCGACGAGAAGACGAA (R)	JCVI_17127
TCCATCCATCGTCCACAG (F) GCATCATCACAAGCATCCTT (R)	Actin of *B. napus*

## Discussion

In this study, complete and stable female sterility in *B. napus* mediated by one alien chromosome was characterized for its anatomy of gynoecium development and gene expressions. The female sterility was insensitive to the dosage of the alien chromosome from *O. violaceus*, because the gynoecium development was nearly the same in the plants with one or two copies of the alien chromosome. This chromosome also caused complete female sterility in the nullisomic line with one chromosome pair lost (2n = 36) of *B. napus* (data unshown), suggesting that the genes on this alien chromosome for female sterility were still active in the altered genetic background of *B. napus*. These results indicated that the female sterile lines were valuable for elucidating the genetic mechanisms behind the gynoecium development for *Brassica* crops and other crucifers.

### Phenotype of aberrant gynoecium

In crucifers, the pollen tube must breach the stigma surface and burrow through the extracellular matrix of the stigma epidermal cells and transmitting tract cells before reaching its ovule targets [34]. It grows typically through the foot to penetrate the stigmatic cuticle and then enters the outer layer of the stigmatic cell wall, which would require cutinase or esterase to break down the stigmatic cuticle and modify the cell walls [35]. In our S1 line, the failure of the pollen tubes to penetrate the stigma epidermal cells on the defective stigmatic papillae may be caused by the aberrant structure and component of the abortive papillae cell walls or the enzyme activities. On the other hand, two differentially expressed unigenes encoded the S locus glycoprotein (SLG) (JCVI_18221 and JCVI_22894) were observed, which were specific to H3 and showed large schange of gene expression level (log2 Ratio(H3/S1) >16). The SLG was expressed specifically in the stigma epidermal cell wall and played a role in pollen-pistil interaction and pollen adhesion [35]. Down-regulated expression of *SLG* was in conformity with that the fewer pollen grains adhered on S1 stigma.

In *Arabidopsis thaliana*, one of several ovule-defective mutants, the *ant* showed ovules without integument development and blocked megasporogenesis at tetrad stage [36]. In ovules of S1 plant, the integuments arrested at the initiative stage and presented a small protuberance at the chalaza region, which indicated that the inner integument could initiate but not grow and the outer integument did not developed. So the abortive stage of S1 was a little later than the *ant* mutant. Consistently, the megasporogenesis was blocked after the tetrad stage and the megagametogenesis did not initiate in S1, as the tetrad and degraded megaspore were observed but the functional megaspore never discovered. Additionally, expression of *ANT* gene showed no difference between S1 and H3, suggesting that defective ovules in S1 may be caused by downstream genes of *ANT* or other metabolic pathways.

### Global gene transcription changes related with female sterility

The fact that more unigenes appeared in S1 (28,065) than H3 (27,653) was obviously connected with the alien chromosomes from *O. violaceus*, suggesting that newly initiated transcription occurred in S1. Furthermore, 4540 DEGs were induced or repressed by more than two folds in S1 plants compared with H3 plants, some of them were confirmed by the qRT-PCR analysis. As there were too many GO enrichment terms when using hypergeometric test for the 4540 unigenes, we chose the DEGs with RPKM ≥ 50 and fold change ≥ 2 (1987) for the GO functional categorization. There were several processes related to female sterile traits, including flower development, embryo sac development, gametophyte development, cell growth, cell differentiation, cell death, cell cycle, pollen-pistil interaction. One inexplicable phenomenon was that, in the ontology of biological process, the most over-represented GO terms were responses to stress or stimulus, suggesting that significant changes of the stress-resistant reaction in female sterile plants happened. More than 200 differentially expressed unigenes without any annotation might have some novel functions.

### Genes and metabolism pathways for female sterility

**Figure 7:**
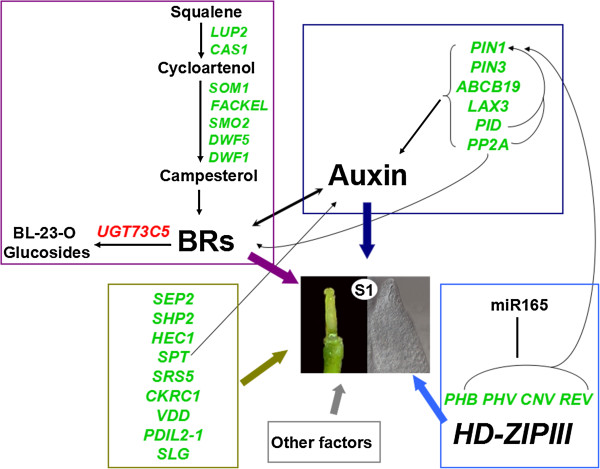
**A proposed model of BR-, auxin-, and HD-ZipIII genes-dependent pathways for gynoecium development.** All genes displayed in this figure correspond to DEGs in this study, other related genes without difference are not showed here. Genes with green color are down-regulated and those with red color are up-regulated in S1 pistils. Different colors of rectangle represent different pathway or factors, purple: BRs synthesis and metabolism, dark blue: polar auxin transport, light blue: HD-ZipIII genes, deep yellow: other related genes. Arrows and bars indicate positive and negative regulatory interactions, respectively. BRs: Brassinosteroids. This figure also includes the information from other sources [3,14,37].

GO and pathway enrichment analysis of the DEGs revealed a number of important genes and metabolism pathways for gynoecium development in S1 plants. The first noticeable pathway was brassinosteroid (BR) biosynthesis and metabolic process. BRs have several physiological effects in plants, including cell elongation and division, vascular differentiation, flowering, pollen development, seed germination and photomorphogenesis [37]. A novel role for BRs in gynoecium and ovule development was shown recently [18]. In our study, twelve differential unigenes were revealed for BR biosynthesis, which encoded seven kinds of proteins (LUP2, CAS1, SMO1, FACKEL, SMO2, DWF5 and DWF1) produced during the biochemical process from squalene to cycloartenol to campesterol to BR (Figure 7). From the pathway of brassinosteroid biosynthesis, all of the twelve unigenes were notably down-regulated in S1 plant and could affect the normal biosynthesis of brassinosteroid. For BR metabolism, only two genes were identified in *Arabidopsis*. One encoded a DON-Glucosyltransferase termed *UGT73C5*, which was found to regulate BR activity by catalyzing the 23-O-glucosylation of BL and castasterone. Overexpression of *UGT73C5* resulted in BR-deficient phenotypes [27]. Another was *BAS1*, which encoded a cytochrome P450 enzyme capable of hydroxylating BR to 26-OH-BL [37]. Here, one unigenes (JCVI_27911) which encoded *UGT73C5* was observed to highly and exclusively expressed in S1 plants (log2 Ratio(S1/H3) >19). Then, potentially reduced BR activity in S1 gynoecia may be the key reason for female sterility.

The second group of genes was those involved in adaxial/abaxial axis specification. Recent studies have shown that adaxial–abaxial polarity mechanisms were required for integument formation [14,38,39]. At present, eight genes from three gene families were confirmed to determine adaxial/abaxial axis specification during the inner and outer integuments development: *INO* from *YABBY* gene family, *KAN1*, *KAN2* and *ABERRANT TESTA SHAPE* (*ATS*, also referred to as *KANADI4*, *KAN4*) from *KANADI* gene family, and four *Class III Homeodomain-leucine zipper* (*HD-ZipIII*) genes (*CNV*, *PHB*, *PHV* and *REV*) [14]. A balance model for the adaxial/abaxial determinants underlying integument morphogenesis was also proposed: in the inner integument, *ATS* and a proposed additional abaxial factor acted in balanced opposition to *CNA/PHB/PHV* and *REV* to promote inner integument growth; in the outer integument, the abaxial activities of *INO*, *KAN1* and *KAN2* acted in balance with *REV* to control morphogenesis [14]. Thus, HD-ZIPIII transcription factors acted in both the outer and inner integuments as adaxial determinants. In our study, thirteen unigenes with differential expression were included in polarity specification of adaxial/abaxial axis. Six of them encoding four kinds of protein: IAMT1 (JCVI_39696), ERECTA (JCVI_34288), HD2 (JCVI_28751, JCVI_5685 and JCVI_29422) and MYB91 (JCVI_11157), were only found to control the development of leaf polarity [40-42], and seven encoding the four HD-ZIPIII transcription factors: PHB (JCVI_16242 and JCVI_36221), PHV (JCVI_19406 and JCVI_29851), CNA (JCVI_25718 and JCVI_10400) and REV (JCVI_32385), had roles in both leaf and integument adaxial/abaxial polarity formation [38]. Mutation of these genes would cause injured ovule integuments and abortive embryo sac. All of the seven unigenes were down-regulated in S1 plants, conforming to the ovule phenotype in S1. This group of genes was possibly the other important factors affecting gynoecium or ovule development in S1. In addition, it has been demonstrated that overexpression of miR165 led to the down-regulation of all five *HD-ZIP III* genes, and concomitantly recapitulated the phenotypes of simultaneous loss-of-function mutation of *REV*, *PHV* and *PHB*[43]. It was possible that the miR165 was overexpressed in gynoecia of S1 and caused the integuments damage indirectly, because the four genes were all down-regulated.

The third related pathway was auxin transport and signaling pathway. As the first plant hormone studied, auxin had wide-ranging effects on growth and development throughout the plant, including gynoecium and ovule morphogenesis [44-46]. Auxin can be distributed by passive diffusion through the mature phloem or active polar auxin transport (called PAT) that mediates cell-to-cell movement of auxin through two different types of proteins, efflux and influx carriers [3]. Transport proteins, best represented by PIN-FORMED1 (PIN1) and P-GLYCOPROTEINS19 (PGP19/ABCB19), have been shown to coordinately regulate auxin efflux, and AUXIN1 (AUX1) and its paralogs LIKE-AUX1 (LAX1-3) are the auxin influx carriers [47]. In our study, 21 unigenes related with auxin transport or signaling pathway showed different expression levels, some encoded the efflux and influx carriers, such as PIN1 (JCVI_31884), PIN3 (JCVI_7893, JCVI_6698 and JCVI_31660), ABCB19 (JCVI_15407 and JCVI_35748) and LAX3 (JCVI_32855), and two PIN phosphorylation regulators PID (JCVI_34187) and PP2A (JCVI_14923). *PIN1*, the first gene in *PIN* gene family, had a role in basipetal auxin transport in stem as a catalytic auxin efflux carrier [3,47]. *PIN3*, another member of *PIN* gene family, was a component of the lateral auxin transport system regulating tropic growth and essentially involved in mediating differential shoot growth [48]. *ABCB19* encoded one of the PGP proteins (PGP19) that belonged to ATP-binding cassette (ABC) transporter superfamily and had an important role in stabilizing PIN1 localization at the plasma membrane microdomains [47]. LAX3, one of the paralogs of AUX1, might function in concentrating auxin in the cytoplasm of cells of L1 layer and preventing auxin diffusion in the apoplast, inducing auxin to flow into the neighboring cells [49]. *PID* encoded a Ser/Thr protein kinase and functioned on PIN phosphorylation that caused the preferential location of PIN in the apical side. But the phosphatase PP2A acted antagonistically to PID on phosphorylation of PIN proteins. Here, all of these genes participating in mediation of auxin fluxes directly or indirectly were down-regulated expressed at different levels. Alteration of these genes probably disturbed the auxin flux and distribution in the S1 gynoecia, which would arrest the normal development of gynoecia and ovules (Figure 7).

The last group of genes was those with important role in gynoecium or ovule development, such as *SHP2*, *HEC1*, *SPT*, *SRS5*, *CKRC1*, *VDD*, *AGO5*, *PDIL2-1*, *SEP2*, *PI*, *AP3*, *EDA14* and *EDA17*. All of these genes were down-regulated except *AGO5*, *PI* and *EDA14*, which was generally in accorded with female sterility. Among these differently expressed genes between S1 and H3, the two MADS box genes, *SHP2* and *SEP2* were reported to be ovule identity factors, controlling ovule integuments identity with other genes [50].

### Interactions between BR, auxin and *HD-ZipIII* genes on gynoecium development

Up to now, numerous studies have addressed that there are interactions and crosstalks between brassinosteroids (BRs) and auxins. It was demonstrated that the IAA genes were induced by brassinolide (BL) via activating the auxin response elements [51]. In the growth of *Arabidopsis* hypocotyl, either application of BRs or disruption of BR synthesis would alter auxin response, presumably by affecting auxin transport [52]. It was confirmed that the gene *BRX* acted at the nexus of a feedback loop that mediates threshold brassinosteroid levels to permit optimal auxin action [53]. A recent study showed that a crucial gene for BR biosynthesis, *DWF4*, played a novel role in the BR-auxin crosstalk [54]. The PP2A protein mediating auxin fluxes mentioned above also had a dual role in the shift between inhibition and activation of BR signaling [55]. Besides BRs, *HD-ZipIII* genes also had the link with auxin. It has been proposed that *IFL1/REV* could influence auxin polar transport and cell differentiation and morphology [56]. The loss of Class III HD-Zip gene activity resulted in a loss of bilateral symmetry during embryogenesis by altering the PIN1 localization and mediating auxin signal transduction [57]. Overall, the brassinosteroid (BR), auxin and *HD-ZipIII* genes likely affected the formation of ovule integuments and growth of gynoecia of S1 plants, respectively and corporately. It has been proposed that the enhanced *seu cyp85A2* double mutant phenotypes in ovule and gynoecium maybe result from the combination of brassinosteroid- and auxin-dependent signaling pathways [18]. Here, a model of BR-, auxin- and *HD-ZipIII* genes-dependent pathways and their interactions for gynoecium development in the female sterile plant was proposed (Figure 7). In addition, the DEGs and related pathways involved in gynoecium development mentioned above were also listed (Additional file 5: Table S4).

## Conclusion

The complete female sterility in *Brassica napus* mediated by one alien chromosome was caused by the lack of formation of inner and outer integuments and the blocked megasporogenesis. Among the 4540 DEGs detected by RNA-seq in the female sterility, a number of important genes and metabolism pathways were involved in the development of gynoecium, ovule, integuments as well as the interactions between pollen and pistil. Particularly, the genes for the pathways related with BR, auxin and adaxial/abaxial axis specification were most likely responsible for the abortive development of female organs.

## Methods

### Plant materials and RNA preparation

The monosomic or disomic additional plants with a single, or one pair of the *Orychophragmus violaceus* chromosomes and all 38 chromosomes of *Brassica napus* (2n = 39, AACC + 1O; 2n = 40, AACC + 2O) were identified among the successive backcrossing progenies of the intergeneric somatic hybrid (2n = 62, AACCOO) with *B. napus* L. cv. Huashuang 3 [26] (Additional file 6: Figure S2, and the method of GISH for Figure S2 was the same as previously described [26]). One of the addition line used in this study was female sterile but male fertile. After the line pollinated *B. napus*, the progenies were found to be male and female fertile *B. napus*, or female sterile *B. napus* with the *O. violaceus* chromosome(s) which were selected for study. The female sterile disomic addition line with one pair of the *O. violaceus* chromosomes (designated as S1) and donor *B. napus* cv. Huashuang No. 3 (H3) were grown in the experimental field of Qinghai University in Xining, Qinghai Province, and gynoecium samples in 1.5 to 3 mm long flower buds were collected. All samples were immediately conserved in RNAfixer Stabilization Reagent (BioTeke Corporation) for one week, then transferred to liquid nitrogen and kept at -80°C until use. Total RNA from the two samples was extracted using the Polysaccharide and Polyphenol Total RNA Isolation Kit (spin column; Bioteke Corporation). The quality of the RNA was analyzed by agarose gel (1.5%) electrophoresis and the total RNA content was assessed by spectroscopy at 260/280 nm (GeneQuant II; Pharmacia Biotech). Finally, the RIN (RNA integrity number) was evaluated using Agilent 2100 by BGI-Shenzhen, and the value of S1 and H3 were 9.2 and 9.1 respectively.

### Pollen germination, pollen tube growth

About 10 pollinated pistils were collected 2, 6, 24, 48 and 72 h after artificial pollinations, and fixed in FAA solution (50% ethanol, 5% acetic acid, 3.7% formaldehyde) at 4°C overnight. Before observation, the pistils were soften with 6 mol/L NaOH for 12 h, then rinsed with distilled water for three times, and dipped in 0.1% water soluble aniline blue solution (aniline blue was diffused in 0.1 mol/l K_3_PO_4_ solution) for 24 h [26]. Then the pistils were mounted on slides and covered lightly with coverslips, and observed under the fluorescence microscopy (Nikon Eclipse 80i).

### Scanning electron microscopy

Fresh gynoecia in flower buds with different sizes and opening flowers of S1 and H3 were collected at the same time, and fixed overnight in 2% glutaraldehyde, and dehydrated through graded ethanol. All samples were dried, sputter-coated and analyzed as previously described [58].

### Whole-mount preparations

Fresh gynoecia in flower buds with different size range and opening flowers of S1 and H3 were collected and fixed in FAA solution at 4°C overnight. After dehydrated through graded ethanol, cleared whole-mount tissues were prepared by dissecting ovules from carpels using needles, and cleared in the mixture with 1/2 Chloral hydrate solution (chloral hydrate:glycerol:water = 8:1:2 ) [59] and 1/2 absolute alcohol for 2 h, and then in Chloral hydrate solution for 3 times (12 h each time). For those too small to dissect ovules, the whole pistils were cleared directly. All samples were observed using a Nikon DS-Ri 1 microscope equipped with differential interface contrast (DIC) optics.

### RNA sequencing library construction, Illumina sequencing, and data processing

Approximately 40 μg total RNA of each sample (S1 and H3) was sent to BGI-Shenzhen where the RNA-seq libraries were constructed and sequenced by using Illumina HiSeq™ 2000. The mRNA enrichment, RNA fragmentation, the first and second strand cDNA synthesis and purifying, sequencing adaptors ligation and PCR amplification were performed as previously described [60]. After sequencing, the preliminary data processing was also carried out by BGI-Shenzhen, according to the procedure. Briefly, the original image data was transferred into sequence data by base calling, which was defined as raw data or raw reads and saved as fastq files. The dirty raw reads including those with adaptors, containing more than 10% of unknown bases, and low quality reads (the percentage of the low quality bases of quality value ≤ 5 is more than 50% in a read) were filtered, then clean reads were obtained for further analysis. *Brassica*_95k_unigene (http://brassica.nbi.ac.uk/) was used as the reference genome, and clean reads of each sample were mapped to reference sequences using SOAP2 [61], allowing no more than two mismatched bases. The gene expression level was calculated by using RPKM method (Reads Per kb per Million reads) [62]. If there were more than one transcript for a gene, the longest one was used to calculate its expression level and coverage.

### Screening of differentially expressed genes (DEGs)

Referring to “The significance of digital gene expression profiles [63]”, BGI have developed a strict algorithm to identify differentially expressed genes between two samples using the Poisson model. Denoted the number of unambiguous clean tags from gene A as x, given that every gene's expression occupies only a small part of the library, p(x) will closely follow the Poisson distribution: p(x) = e^-λ^λ^x^ / x! (λ is the real transcripts of the gene). Then, P-value corresponds to differential gene expression test. FDR (False Discovery Rate) is a method to determine the threshold of P-value in multiple tests. More stringent criteria with smaller FDR and bigger fold-change value can be used to identify DEGs. We used “FDR ≤ 0.001 and the absolute value of log_2_Ratio ≥ 1” as the threshold to judge the significance of gene expression difference.

### GO and pathway enrichment analysis of DEGs

GO enrichment analysis provides all GO terms that are significantly enriched in DEGs comparing to the genome background, and filter the DEGs that correspond to biological functions. This method firstly maps all DEGs to GO terms in the database, calculating gene numbers for every term, then using hypergeometric test to find significantly enriched GO terms in DEGs comparing to the genome background. The calculating formula used was the same as previously described [64]. The calculated p-value goes through Bonferroni Correction, taking corrected p-value ≤ 0.05 as a threshold. GO terms fulfilling this condition are defined as significantly enriched GO terms in DEGs. Pathway enrichment analysis also applies a hypergeometric test to identify significantly enriched metabolic pathways or signal transduction pathways in DEGs comparing with the whole genome background, using the major public pathway-related database: KEGG [65]. The calculating formula is the same as that in GO analysis, the pathways with a Q value of ≤ 0.05 are defined as those with significantly differentially expressed (enriched) genes [64]. Additionally, GO function analysis of DEGs with the criteria of RPKM ≥ 50 and fold change ≥ 2, DEGs specific to S1 and specific to H3 plants were performed using blast2Go (http://www.blast2go.com/b2ghome), respectively. GO enrichment analysis of these genes was performed based on the TAIR GO slim provided by blast2GO, filtered by Seq: cutoff = 5.0, as well as the soft agriGO (http://bioinfo.cau.edu.cn/agriGO/).

### Real-time Quantitative RT-PCR (qRT-PCR) analysis

The RNA samples used for the qRT-PCR assays were the same as for the RNA-seq experiments. First-strand cDNA synthesis was performed with 1500 ng of total RNA using RevertAid™ First Strand cDNA Synthesis Kit (Fermentas), total RNA (0.5 μg) was reverse-transcribed with oligo (dT)_18_ primer (0.5 μg/μl) using RevertAid™ Reverse Transcriptase according to the described protocol. Gene-specific primers were designed according to the reference unigene sequences using the Primer 3.0, all primer sequences are listed in Table 3. A primer was also designed for *B. napus* actin gene to normalize the amplification efficiency. QRT-PCR assays in triplicate were performed using THUNDERBIRD SYBR qPCR Mix (Toyobo, Osaka, Japan) with a Bio-Rad CFX96 Real-Time Detection System. The actin gene was used as an internal control for data normalization, and quantitative variation in the different replicates was calculated using the delta-delta threshold cycle relative quantification method.

### Availability of supporting data

The data set supporting the results of this article is available in NCBI’s Gene Expression Omnibus (http://www.ncbi.nlm.nih.gov/geo/) under accession number GSE49606 (http://www.ncbi.nlm.nih.gov/geo/query/acc.cgi?acc=GSE49606). Other supporting data are included within the article and its additional files.

## Competing interests

The authors declare that they have no competing interests.

## Authors’ contributions

ZYL and WQF conceived of the study. WQF carried out the anatomical and molecular woks. ZGZ and LD obtained the female sterile addition line. WQF, ZYL and XHG analyzed the data and drafted the manuscript. All authors have read and approved the final manuscript.

## Supplementary Material

Additional file 1: Figure S1 Morphology of different-stage flower buds and corresponding pistils of H3 (**A** and **C**) and S1 (**B** and **D**). Numbers above: length of flower buds, numbers below: length of corresponding pistils, unit: mm. Bar = 1 cm. Click here for file

Additional file 2: Table S1 Significantly enriched GO terms in the 4540 DEGs according to hypergeometric test. Click here for file

Additional file 3: Table S2 Significantly enriched GO terms in the DEGs specific to S1. Click here for file

Additional file 4: Table S3 Significantly enriched GO terms in the DEGs specific to H3. Click here for file

Additional file 5: Table S4 List of DEGs and related pathways involved in gynoecium development based on KEGG pathway and biological process of GO. Click here for file

Additional file 6: Figure S2 GISH analysis of the monosomic **(A)** and disomic **(B)** additional plants. Red signals are from the labeled *O.violaceus* probe and blue color from DAPI staining. In PMCs at meiotic anaphase I, the chromosomes show the 19:20 and 19:21 segregations with one **(A)** and two **(B)** labeled chromosomes, respectively. Note that the two chromosomes from *O. violaceus* move to one polar group, other than equally segregate. Bar = 5 μm. Click here for file
