# Supplementary figures and images for: Anatomy and transcript profiling of gynoecium development in female sterile Brassica napus mediated by one alien chromosome from Orychophragmus violaceus
Source: BMC Genomics. 2014 Jan 23;15:61. doi: 10.1186/1471-2164-15-61 (PMC3930543; doi:10.1186/1471-2164-15-61)

**1 2 3 4 5 6 7 8 9 1 2 3 4 5 6 7 8 9**

**0.8 1.5 2.0 3.0 3.8 4.5 4.5 5.0 6.0 0.8 1.2 1.6 1.8 1.8 1.8 1.8 2.0 2.5**


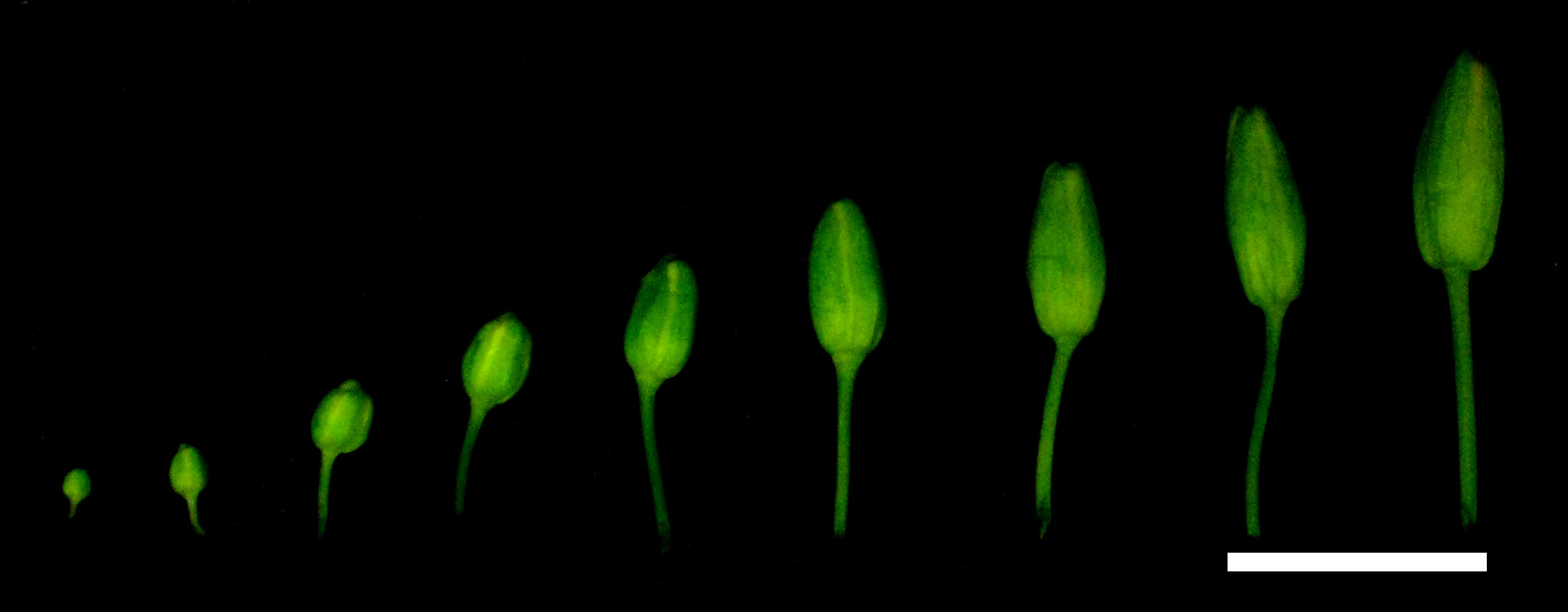


**A**


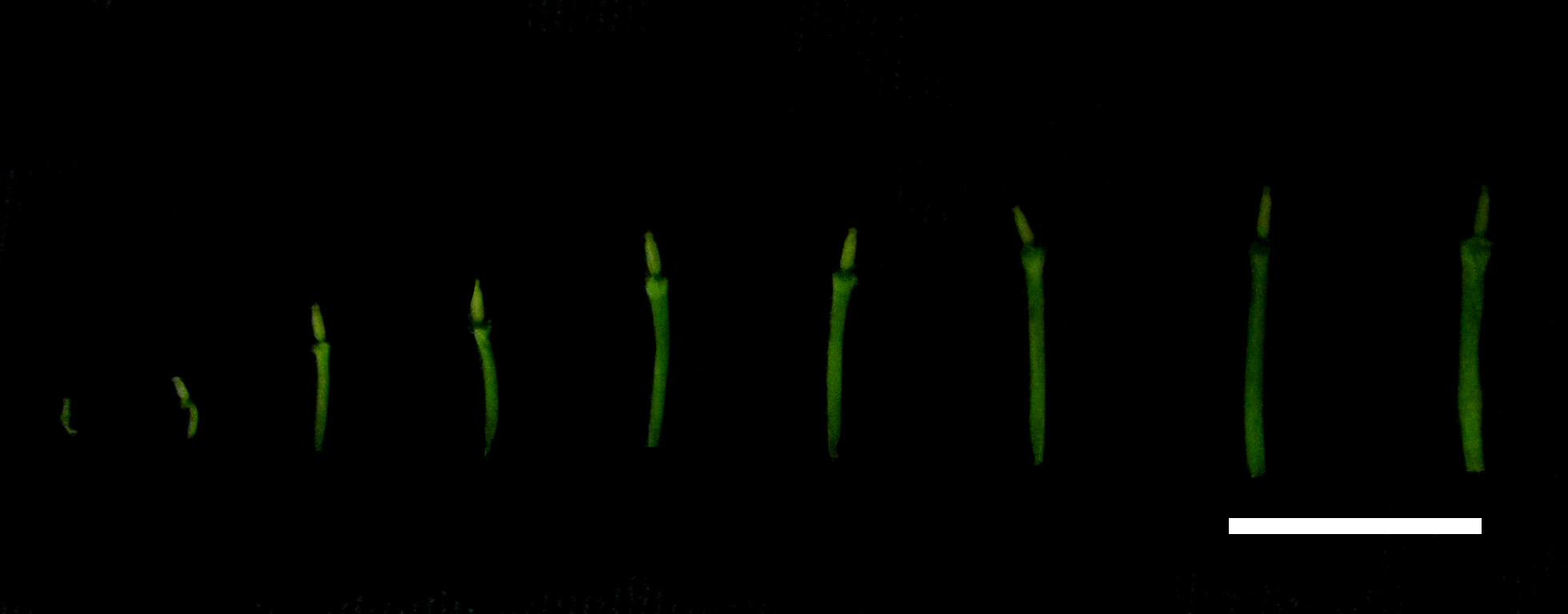


**D**


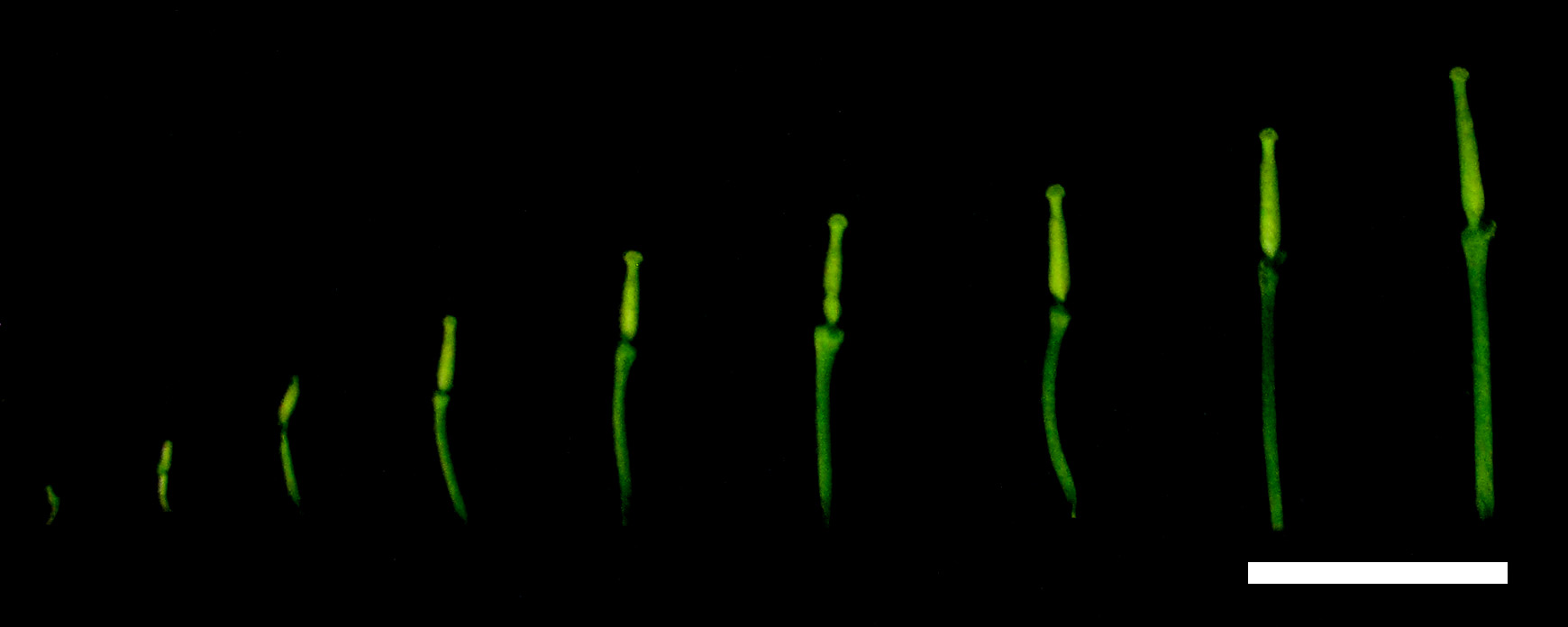


**C**


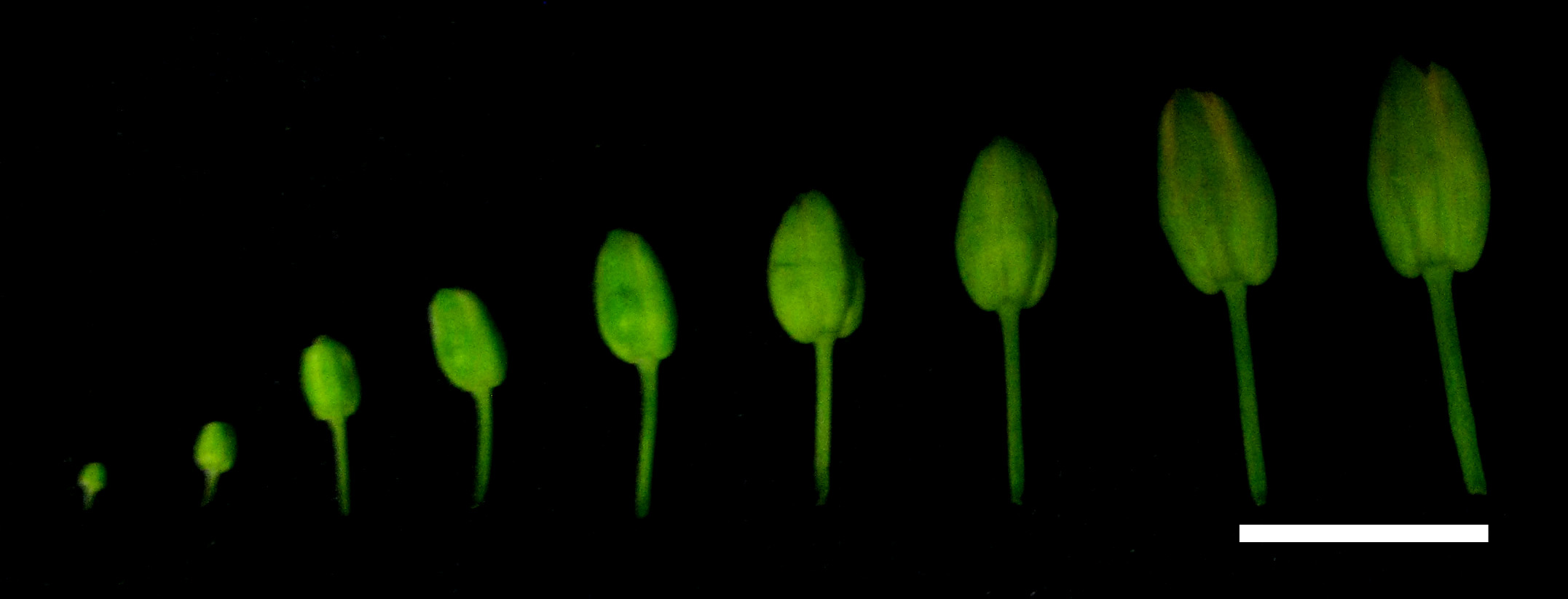


**B**

Supplement: Additional file 1: Figure S1 — Morphology of different-stage flower buds and corresponding pistils of H3 (A and C) and S1 (B and D). Numbers above: length of flower buds, numbers below: length of corresponding pistils, unit: mm. Bar = 1 cm. [file 1471-2164-15-61-S1.doc]
