# Supplementary material for: Anatomy and transcript profiling of gynoecium development in female sterile Brassica napus mediated by one alien chromosome from Orychophragmus violaceus
Source: BMC Genomics. 2014 Jan 23;15:61. doi: 10.1186/1471-2164-15-61 (PMC3930543; doi:10.1186/1471-2164-15-61)
Supplement: Additional file 2: Table S1 — Significantly enriched GO terms in the 4540 DEGs according to hypergeometric test. [file 1471-2164-15-61-S2.doc]

| **Gene Ontology term** | **Term type^a^** | **Cluster frequency** | **Genome frequency of use** | **Corrected P-value^b^** |
| --- | --- | --- | --- | --- |
| [External encapsulating structure](http://amigo.geneontology.org/cgi-bin/amigo/go.cgi?action=query&view=query&query=GO:0030312&search_constraint=terms) | C | 303 out of 2631 (11.5%) | 2363 out of 34440 (6.9%) | 1.00e-17 |
| [Cell periphery](http://amigo.geneontology.org/cgi-bin/amigo/go.cgi?action=query&view=query&query=GO:0071944&search_constraint=terms) | C | 328 out of 2631 (12.5%) | 2721 out of 34440 (7.9%) | 2.97e-15 |
| [Cell wall](http://amigo.geneontology.org/cgi-bin/amigo/go.cgi?action=query&view=query&query=GO:0005618&search_constraint=terms) | C | 161 out of 2631 (6.1%) | 1058 out of 34440 (3.1%) | 3.68e-15 |
| [Extracellular region](http://amigo.geneontology.org/cgi-bin/amigo/go.cgi?action=query&view=query&query=GO:0005576&search_constraint=terms) | C | 202 out of 2631 (7.7%) | 1461 out of 34440 (4.2%) | 1.22e-14 |
| [Plastid](http://amigo.geneontology.org/cgi-bin/amigo/go.cgi?action=query&view=query&query=GO:0009536&search_constraint=terms) | C | 648 out of 2631 (24.6%) | 7111 out of 34440 (20.6%) | 2.35e-05 |
| [Cytoplasmic part](http://amigo.geneontology.org/cgi-bin/amigo/go.cgi?action=query&view=query&query=GO:0044444&search_constraint=terms) | C | 1102 out of 2631 (41.9%) | 12847 out of 34440 (37.3%) | 4.46e-05 |
| [Cytoplasm](http://amigo.geneontology.org/cgi-bin/amigo/go.cgi?action=query&view=query&query=GO:0005737&search_constraint=terms) | C | 1105 out of 2631 (42.0%) | 12894 out of 34440 (37.4%) | 5.15e-05 |
| [Plastid part](http://amigo.geneontology.org/cgi-bin/amigo/go.cgi?action=query&view=query&query=GO:0044435&search_constraint=terms) | C | 309 out of 2631 (11.7%) | 3111 out of 34440 (9.0%) | 0.00010 |
| [Organelle part](http://amigo.geneontology.org/cgi-bin/amigo/go.cgi?action=query&view=query&query=GO:0044422&search_constraint=terms) | C | 691 out of 2631 (26.3%) | 7724 out of 34440 (22.4%) | 0.00012 |
| [Intracellular organelle part](http://amigo.geneontology.org/cgi-bin/amigo/go.cgi?action=query&view=query&query=GO:0044446&search_constraint=terms) | C | 587 out of 2631 (22.3%) | 6465 out of 34440 (18.8%) | 0.00018 |
| [Nuclear lumen](http://amigo.geneontology.org/cgi-bin/amigo/go.cgi?action=query&view=query&query=GO:0031981&search_constraint=terms) | C | 156 out of 2631 (5.9%) | 1427 out of 34440 (4.1%) | 0.00055 |
| [Organelle lumen](http://amigo.geneontology.org/cgi-bin/amigo/go.cgi?action=query&view=query&query=GO:0043233&search_constraint=terms) | C | 156 out of 2631 (5.9%) | 1427 out of 34440 (4.1%) | 0.00055 |
| [Intracellular organelle lumen](http://amigo.geneontology.org/cgi-bin/amigo/go.cgi?action=query&view=query&query=GO:0070013&search_constraint=terms) | C | 156 out of 2631 (5.9%) | 1427 out of 34440 (4.1%) | 0.00055 |
| [Membrane-enclosed lumen](http://amigo.geneontology.org/cgi-bin/amigo/go.cgi?action=query&view=query&query=GO:0031974&search_constraint=terms) | C | 157 out of 2631 (6.0%) | 1444 out of 34440 (4.2%) | 0.00070 |
| [Chloroplast thylakoid](http://amigo.geneontology.org/cgi-bin/amigo/go.cgi?action=query&view=query&query=GO:0009534&search_constraint=terms) | C | 113 out of 2631 (4.3%) | 983 out of 34440 (2.9%) | 0.00140 |
| [Plastid thylakoid](http://amigo.geneontology.org/cgi-bin/amigo/go.cgi?action=query&view=query&query=GO:0031976&search_constraint=terms) | C | 119 out of 2631 (4.5%) | 1052 out of 34440 (3.1%) | 0.00173 |
| [Organelle subcompartment](http://amigo.geneontology.org/cgi-bin/amigo/go.cgi?action=query&view=query&query=GO:0031984&search_constraint=terms) | C | 120 out of 2631 (4.6%) | 1064 out of 34440 (3.1%) | 0.00183 |
| [Photosystem](http://amigo.geneontology.org/cgi-bin/amigo/go.cgi?action=query&view=query&query=GO:0009521&search_constraint=terms) | C | 31 out of 2631 (1.2%) | 180 out of 34440 (0.5%) | 0.00263 |
| [Anchored to membrane](http://amigo.geneontology.org/cgi-bin/amigo/go.cgi?action=query&view=query&query=GO:0031225&search_constraint=terms) | C | 36 out of 2631 (1.4%) | 227 out of 34440 (0.7%) | 0.00377 |
| [Thylakoid](http://amigo.geneontology.org/cgi-bin/amigo/go.cgi?action=query&view=query&query=GO:0009579&search_constraint=terms) | C | 127 out of 2631 (4.8%) | 1177 out of 34440 (3.4%) | 0.00801 |
| [Organelle](http://amigo.geneontology.org/cgi-bin/amigo/go.cgi?action=query&view=query&query=GO:0043226&search_constraint=terms) | C | 1900 out of 2631 (72.2%) | 23815 out of 34440 (69.1%) | 0.03055 |
| [Photosynthetic membrane](http://amigo.geneontology.org/cgi-bin/amigo/go.cgi?action=query&view=query&query=GO:0034357&search_constraint=terms) | C | 39 out of 2631 (1.5%) | 281 out of 34440 (0.8%) | 0.03467 |
| [Organelle envelope](http://amigo.geneontology.org/cgi-bin/amigo/go.cgi?action=query&view=query&query=GO:0031967&search_constraint=terms) | C | 226 out of 2631 (8.6%) | 2370 out of 34440 (6.9%) | 0.04310 |
| [Thylakoid part](http://amigo.geneontology.org/cgi-bin/amigo/go.cgi?action=query&view=query&query=GO:0044436&search_constraint=terms) | C | 48 out of 2631 (1.8%) | 374 out of 34440 (1.1%) | 0.04694 |
| [Nuclear part](http://amigo.geneontology.org/cgi-bin/amigo/go.cgi?action=query&view=query&query=GO:0044428&search_constraint=terms) | C | 158 out of 2631 (6.0%) | 1582 out of 34440 (4.6%) | 0.04911 |
| [Oxidoreductase activity](http://amigo.geneontology.org/cgi-bin/amigo/go.cgi?action=query&view=query&query=GO:0016491&search_constraint=terms) | F | 425 out of 2745 (15.5%) | 3938 out of 36590 (10.8%) | 7.85e-13 |
| [Oxidoreductase activity, acting on the CH-CH group of donors, NAD or NADP as acceptor](http://amigo.geneontology.org/cgi-bin/amigo/go.cgi?action=query&view=query&query=GO:0016628&search_constraint=terms) | F | 76 out of 2745 (2.8%) | 496 out of 36590 (1.4%) | 9.44e-07 |
| [Oxidoreductase activity, acting on the CH-CH group of donors](http://amigo.geneontology.org/cgi-bin/amigo/go.cgi?action=query&view=query&query=GO:0016627&search_constraint=terms) | F | 88 out of 2745 (3.2%) | 616 out of 36590 (1.7%) | 1.81e-06 |
| [Oxidoreductase activity, acting on single donors with incorporation of molecular oxygen, incorporation of two atoms of oxygen](http://amigo.geneontology.org/cgi-bin/amigo/go.cgi?action=query&view=query&query=GO:0016702&search_constraint=terms) | F | 19 out of 2745 (0.7%) | 78 out of 36590 (0.2%) | 0.00150 |
| [Dioxygenase activity](http://amigo.geneontology.org/cgi-bin/amigo/go.cgi?action=query&view=query&query=GO:0051213&search_constraint=terms) | F | 19 out of 2745 (0.7%) | 78 out of 36590 (0.2%) | 0.00150 |
| [Lyase activity](http://amigo.geneontology.org/cgi-bin/amigo/go.cgi?action=query&view=query&query=GO:0016829&search_constraint=terms) | F | 115 out of 2745 (4.2%) | 1019 out of 36590 (2.8%) | 0.00320 |
| [Peptide binding](http://amigo.geneontology.org/cgi-bin/amigo/go.cgi?action=query&view=query&query=GO:0042277&search_constraint=terms) | F | 18 out of 2745 (0.7%) | 80 out of 36590 (0.2%) | 0.00871 |
| [Isoprenoid binding](http://amigo.geneontology.org/cgi-bin/amigo/go.cgi?action=query&view=query&query=GO:0019840&search_constraint=terms) | F | 8 out of 2745 (0.3%) | 19 out of 36590 (0.1%) | 0.01467 |
| [Glyceraldehyde-3-phosphate dehydrogenase activity](http://amigo.geneontology.org/cgi-bin/amigo/go.cgi?action=query&view=query&query=GO:0008943&search_constraint=terms) | F | 12 out of 2745 (0.4%) | 42 out of 36590 (0.1%) | 0.01696 |
| [Hydrolase activity, hydrolyzing O-glycosyl compounds](http://amigo.geneontology.org/cgi-bin/amigo/go.cgi?action=query&view=query&query=GO:0004553&search_constraint=terms) | F | 93 out of 2745 (3.4%) | 824 out of 36590 (2.3%) | 0.02284 |
| [Gibberellin 2-beta-dioxygenase activity](http://amigo.geneontology.org/cgi-bin/amigo/go.cgi?action=query&view=query&query=GO:0045543&search_constraint=terms) | F | 6 out of 2745 (0.2%) | 11 out of 36590 (0.0%) | 0.02443 |
| [Oxidoreductase activity, acting on the aldehyde or oxo group of donors, NAD or NADP as acceptor](http://amigo.geneontology.org/cgi-bin/amigo/go.cgi?action=query&view=query&query=GO:0016620&search_constraint=terms) | F | 37 out of 2745 (1.3%) | 251 out of 36590 (0.7%) | 0.02612 |
| [Oxidoreductase activity, acting on single donors with incorporation of molecular oxygen](http://amigo.geneontology.org/cgi-bin/amigo/go.cgi?action=query&view=query&query=GO:0016701&search_constraint=terms) | F | 28 out of 2745 (1.0%) | 170 out of 36590 (0.5%) | 0.02840 |
| [Alpha-glucosidase activity](http://amigo.geneontology.org/cgi-bin/amigo/go.cgi?action=query&view=query&query=GO:0004558&search_constraint=terms) | F | 9 out of 2745 (0.3%) | 27 out of 36590 (0.1%) | 0.04160 |
| [Response to stimulus](http://amigo.geneontology.org/cgi-bin/amigo/go.cgi?action=query&view=query&query=GO:0050896&search_constraint=terms) | P | 1214 out of 2674 (45.4%) | 12045 out of 34074 (35.3%) | 4.58e-26 |
| [Response to chemical stimulus](http://amigo.geneontology.org/cgi-bin/amigo/go.cgi?action=query&view=query&query=GO:0042221&search_constraint=terms) | P | 678 out of 2674 (25.4%) | 6110 out of 34074 (17.9%) | 1.01e-20 |
| [Response to abiotic stimulus](http://amigo.geneontology.org/cgi-bin/amigo/go.cgi?action=query&view=query&query=GO:0009628&search_constraint=terms) | P | 512 out of 2674 (19.1%) | 4424 out of 34074 (13.0%) | 4.33e-18 |
| [Response to stress](http://amigo.geneontology.org/cgi-bin/amigo/go.cgi?action=query&view=query&query=GO:0006950&search_constraint=terms) | P | 689 out of 2674 (25.8%) | 6562 out of 34074 (19.3%) | 5.21e-15 |
| [Response to inorganic substance](http://amigo.geneontology.org/cgi-bin/amigo/go.cgi?action=query&view=query&query=GO:0010035&search_constraint=terms) | P | 283 out of 2674 (10.6%) | 2225 out of 34074 (6.5%) | 1.30e-13 |
| [Response to metal ion](http://amigo.geneontology.org/cgi-bin/amigo/go.cgi?action=query&view=query&query=GO:0010038&search_constraint=terms) | P | 242 out of 2674 (9.1%) | 1884 out of 34074 (5.5%) | 8.84e-12 |
| [Response to radiation](http://amigo.geneontology.org/cgi-bin/amigo/go.cgi?action=query&view=query&query=GO:0009314&search_constraint=terms) | P | 230 out of 2674 (8.6%) | 1820 out of 34074 (5.3%) | 2.41e-10 |
| [Secondary metabolic process](http://amigo.geneontology.org/cgi-bin/amigo/go.cgi?action=query&view=query&query=GO:0019748&search_constraint=terms) | P | 166 out of 2674 (6.2%) | 1267 out of 34074 (3.7%) | 3.89e-08 |
| [Response to carbohydrate stimulus](http://amigo.geneontology.org/cgi-bin/amigo/go.cgi?action=query&view=query&query=GO:0009743&search_constraint=terms) | P | 121 out of 2674 (4.5%) | 842 out of 34074 (2.5%) | 6.62e-08 |
| [Response to reactive oxygen species](http://amigo.geneontology.org/cgi-bin/amigo/go.cgi?action=query&view=query&query=GO:0000302&search_constraint=terms) | P | 48 out of 2674 (1.8%) | 229 out of 34074 (0.7%) | 3.05e-07 |
| [Response to light stimulus](http://amigo.geneontology.org/cgi-bin/amigo/go.cgi?action=query&view=query&query=GO:0009416&search_constraint=terms) | P | 170 out of 2674 (6.4%) | 1346 out of 34074 (4.0%) | 3.95e-07 |
| [Response to organic substance](http://amigo.geneontology.org/cgi-bin/amigo/go.cgi?action=query&view=query&query=GO:0010033&search_constraint=terms) | P | 411 out of 2674 (15.4%) | 3944 out of 34074 (11.6%) | 4.60e-07 |
| [Response to osmotic stress](http://amigo.geneontology.org/cgi-bin/amigo/go.cgi?action=query&view=query&query=GO:0006970&search_constraint=terms) | P | 215 out of 2674 (8.0%) | 1843 out of 34074 (5.4%) | 2.17e-06 |
| [Cellular aromatic compound metabolic process](http://amigo.geneontology.org/cgi-bin/amigo/go.cgi?action=query&view=query&query=GO:0006725&search_constraint=terms) | P | 136 out of 2674 (5.1%) | 1043 out of 34074 (3.1%) | 3.21e-06 |
| [Toxin metabolic process](http://amigo.geneontology.org/cgi-bin/amigo/go.cgi?action=query&view=query&query=GO:0009404&search_constraint=terms) | P | 26 out of 2674 (1.0%) | 96 out of 34074 (0.3%) | 1.49e-05 |
| [Response to light intensity](http://amigo.geneontology.org/cgi-bin/amigo/go.cgi?action=query&view=query&query=GO:0009642&search_constraint=terms) | P | 43 out of 2674 (1.6%) | 223 out of 34074 (0.7%) | 3.39e-05 |
| [Response to oxidative stress](http://amigo.geneontology.org/cgi-bin/amigo/go.cgi?action=query&view=query&query=GO:0006979&search_constraint=terms) | P | 52 out of 2674 (1.9%) | 305 out of 34074 (0.9%) | 9.50e-05 |
| [Aromatic compound biosynthetic process](http://amigo.geneontology.org/cgi-bin/amigo/go.cgi?action=query&view=query&query=GO:0019438&search_constraint=terms) | P | 71 out of 2674 (2.7%) | 478 out of 34074 (1.4%) | 0.00015 |
| [Phenylpropanoid metabolic process](http://amigo.geneontology.org/cgi-bin/amigo/go.cgi?action=query&view=query&query=GO:0009698&search_constraint=terms) | P | 72 out of 2674 (2.7%) | 494 out of 34074 (1.4%) | 0.00027 |
| [Aromatic amino acid family metabolic process](http://amigo.geneontology.org/cgi-bin/amigo/go.cgi?action=query&view=query&query=GO:0009072&search_constraint=terms) | P | 29 out of 2674 (1.1%) | 138 out of 34074 (0.4%) | 0.00088 |
| [Cellular amino acid metabolic process](http://amigo.geneontology.org/cgi-bin/amigo/go.cgi?action=query&view=query&query=GO:0006520&search_constraint=terms) | P | 139 out of 2674 (5.2%) | 1186 out of 34074 (3.5%) | 0.00127 |
| [Response to UV](http://amigo.geneontology.org/cgi-bin/amigo/go.cgi?action=query&view=query&query=GO:0009411&search_constraint=terms) | P | 45 out of 2674 (1.7%) | 270 out of 34074 (0.8%) | 0.00128 |
| [Phenylpropanoid biosynthetic process](http://amigo.geneontology.org/cgi-bin/amigo/go.cgi?action=query&view=query&query=GO:0009699&search_constraint=terms) | P | 62 out of 2674 (2.3%) | 423 out of 34074 (1.2%) | 0.00147 |
| [Response to endogenous stimulus](http://amigo.geneontology.org/cgi-bin/amigo/go.cgi?action=query&view=query&query=GO:0009719&search_constraint=terms) | P | 344 out of 2674 (12.9%) | 3468 out of 34074 (10.2%) | 0.00205 |
| [Fatty acid biosynthetic process](http://amigo.geneontology.org/cgi-bin/amigo/go.cgi?action=query&view=query&query=GO:0006633&search_constraint=terms) | P | 43 out of 2674 (1.6%) | 261 out of 34074 (0.8%) | 0.00297 |
| [Amine metabolic process](http://amigo.geneontology.org/cgi-bin/amigo/go.cgi?action=query&view=query&query=GO:0009308&search_constraint=terms) | P | 153 out of 2674 (5.7%) | 1357 out of 34074 (4.0%) | 0.00347 |
| [Cellular amine metabolic process](http://amigo.geneontology.org/cgi-bin/amigo/go.cgi?action=query&view=query&query=GO:0044106&search_constraint=terms) | P | 145 out of 2674 (5.4%) | 1274 out of 34074 (3.7%) | 0.00379 |
| [Cellular ketone metabolic process](http://amigo.geneontology.org/cgi-bin/amigo/go.cgi?action=query&view=query&query=GO:0042180&search_constraint=terms) | P | 260 out of 2674 (9.7%) | 2560 out of 34074 (7.5%) | 0.00776 |
| [Oxoacid metabolic process](http://amigo.geneontology.org/cgi-bin/amigo/go.cgi?action=query&view=query&query=GO:0043436&search_constraint=terms) | P | 258 out of 2674 (9.6%) | 2541 out of 34074 (7.5%) | 0.00860 |
| [Carboxylic acid metabolic process](http://amigo.geneontology.org/cgi-bin/amigo/go.cgi?action=query&view=query&query=GO:0019752&search_constraint=terms) | P | 257 out of 2674 (9.6%) | 2531 out of 34074 (7.4%) | 0.00892 |
| [Organic acid metabolic process](http://amigo.geneontology.org/cgi-bin/amigo/go.cgi?action=query&view=query&query=GO:0006082&search_constraint=terms) | P | 258 out of 2674 (9.6%) | 2546 out of 34074 (7.5%) | 0.00991 |
| [Carbohydrate catabolic process](http://amigo.geneontology.org/cgi-bin/amigo/go.cgi?action=query&view=query&query=GO:0016052&search_constraint=terms) | P | 72 out of 2674 (2.7%) | 545 out of 34074 (1.6%) | 0.01005 |
| [Indole-containing compound metabolic process](http://amigo.geneontology.org/cgi-bin/amigo/go.cgi?action=query&view=query&query=GO:0042430&search_constraint=terms) | P | 26 out of 2674 (1.0%) | 135 out of 34074 (0.4%) | 0.01670 |
| [Glucose metabolic process](http://amigo.geneontology.org/cgi-bin/amigo/go.cgi?action=query&view=query&query=GO:0006006&search_constraint=terms) | P | 54 out of 2674 (2.0%) | 381 out of 34074 (1.1%) | 0.01798 |
| [Glucose catabolic process](http://amigo.geneontology.org/cgi-bin/amigo/go.cgi?action=query&view=query&query=GO:0006007&search_constraint=terms) | P | 47 out of 2674 (1.8%) | 320 out of 34074 (0.9%) | 0.02463 |
| [Hexose metabolic process](http://amigo.geneontology.org/cgi-bin/amigo/go.cgi?action=query&view=query&query=GO:0019318&search_constraint=terms) | P | 68 out of 2674 (2.5%) | 524 out of 34074 (1.5%) | 0.03104 |
| [Hexose catabolic process](http://amigo.geneontology.org/cgi-bin/amigo/go.cgi?action=query&view=query&query=GO:0019320&search_constraint=terms) | P | 47 out of 2674 (1.8%) | 324 out of 34074 (1.0%) | 0.03387 |
| [Monosaccharide catabolic process](http://amigo.geneontology.org/cgi-bin/amigo/go.cgi?action=query&view=query&query=GO:0046365&search_constraint=terms) | P | 47 out of 2674 (1.8%) | 324 out of 34074 (1.0%) | 0.03387 |
| [Alcohol catabolic process](http://amigo.geneontology.org/cgi-bin/amigo/go.cgi?action=query&view=query&query=GO:0046164&search_constraint=terms) | P | 47 out of 2674 (1.8%) | 325 out of 34074 (1.0%) | 0.03664 |

^a^ GO term classifications: P, Biological Process; C, Cellular Component; F, Molecular Function.

^b^ GO terms with P-value<0.05 were regarded as significantly enriched GO terms.
