# Supplementary material for: Anatomy and transcript profiling of gynoecium development in female sterile Brassica napus mediated by one alien chromosome from Orychophragmus violaceus
Source: BMC Genomics. 2014 Jan 23;15:61. doi: 10.1186/1471-2164-15-61 (PMC3930543; doi:10.1186/1471-2164-15-61)
Supplement: Additional file 3: Table S2 — Significantly enriched GO terms in the DEGs specific to S1. [file 1471-2164-15-61-S3.doc]

| **GO term description** | **Ontology^a^** | **Number in input list** | **Number in BG/Ref** | **p-value** | **FDR^b^** |
| --- | --- | --- | --- | --- | --- |
| Response to abiotic stimulus | P | 33 | 1471 | 9.10E-16 | 1.70E-13 |
| Response to stress | P | 38 | 2320 | 1.40E-13 | 1.30E-11 |
| Secondary metabolic process | P | 18 | 489 | 5.10E-13 | 3.10E-11 |
| Response to stimulus | P | 50 | 4057 | 1.80E-12 | 6.70E-11 |
| Cellular amino acid and derivative metabolic process | P | 20 | 682 | 1.80E-12 | 6.70E-11 |
| Response to external stimulus | P | 12 | 429 | 5.90E-08 | 1.80E-06 |
| Metabolic process | P | 78 | 10614 | 9.50E-08 | 2.30E-06 |
| Response to biotic stimulus | P | 14 | 638 | 1.00E-07 | 2.30E-06 |
| Cellular process | P | 79 | 11684 | 1.30E-06 | 2.60E-05 |
| Generation of precursor metabolites and energy | P | 8 | 285 | 8.30E-06 | 0.00015 |
| Post-embryonic development | P | 12 | 705 | 9.70E-06 | 0.00016 |
| Primary metabolic process | P | 59 | 8995 | 2.80E-05 | 0.00042 |
| Cellular metabolic process | P | 56 | 8722 | 6.50E-05 | 0.00091 |
| Biosynthetic process | P | 36 | 5118 | 0.00016 | 0.0021 |
| Transcription | P | 18 | 1923 | 0.00022 | 0.0026 |
| Catabolic process | P | 14 | 1307 | 0.00027 | 0.003 |
| Multicellular organismal development | P | 18 | 2020 | 0.00038 | 0.0041 |
| Multicellular organismal process | P | 18 | 2094 | 0.00057 | 0.0058 |
| Nucleobase, nucleoside, nucleotide and nucleic acid metabolic process | P | 24 | 3198 | 0.0006 | 0.0058 |
| Response to endogenous stimulus | P | 11 | 1068 | 0.0015 | 0.013 |
| Developmental process | P | 18 | 2304 | 0.0016 | 0.014 |
| Nitrogen compound metabolic process | P | 24 | 3826 | 0.0053 | 0.044 |
| Binding | F | 70 | 11258 | 3.50E-05 | 0.0015 |
| Catalytic activity | F | 56 | 9638 | 0.00056 | 0.012 |
| Nucleotide binding | F | 17 | 2267 | 0.0031 | 0.042 |
| Nucleoplasm | C | 15 | 177 | 2.20E-16 | 2.50E-14 |
| Nuclear lumen | C | 18 | 374 | 5.60E-15 | 3.20E-13 |
| Intracellular organelle lumen | C | 18 | 539 | 2.50E-12 | 5.90E-11 |
| Membrane-enclosed lumen | C | 18 | 546 | 3.10E-12 | 5.90E-11 |
| Nuclear part | C | 18 | 543 | 2.80E-12 | 5.90E-11 |
| Organelle lumen | C | 18 | 539 | 2.50E-12 | 5.90E-11 |
| Vacuole | C | 15 | 383 | 1.60E-11 | 2.60E-10 |
| Plasma membrane | C | 25 | 1456 | 3.70E-10 | 5.40E-09 |
| Membrane-bounded organelle | C | 61 | 7622 | 8.80E-08 | 1.00E-06 |
| Intracellular membrane-bounded organelle | C | 61 | 7615 | 8.50E-08 | 1.00E-06 |
| Intracellular organelle | C | 62 | 8149 | 3.40E-07 | 3.30E-06 |
| Organelle | C | 62 | 8155 | 3.40E-07 | 3.30E-06 |
| Intracellular | C | 70 | 9671 | 4.60E-07 | 3.80E-06 |
| Intracellular part | C | 68 | 9302 | 4.50E-07 | 3.80E-06 |
| Nucleus | C | 28 | 2621 | 6.70E-07 | 5.10E-06 |
| Membrane | C | 35 | 4068 | 4.40E-06 | 3.20E-05 |
| Cell | C | 94 | 15217 | 6.20E-06 | 4.20E-05 |
| Cytoplasm | C | 50 | 6822 | 6.50E-06 | 4.20E-05 |
| Cytoplasmic part | C | 44 | 6289 | 4.80E-05 | 0.00029 |
| Cell part | C | 86 | 15217 | 0.00014 | 0.00081 |
| Extracellular region | C | 6 | 285 | 0.0005 | 0.0027 |
| Cell wall | C | 7 | 403 | 0.00055 | 0.0029 |
| External encapsulating structure | C | 7 | 407 | 0.00059 | 0.0029 |
| Plastid | C | 22 | 2965 | 0.0011 | 0.0052 |
| Intracellular organelle part | C | 18 | 2561 | 0.0046 | 0.02 |
| Organelle part | C | 18 | 2562 | 0.0046 | 0.02 |

^a^ GO term classifications: P, Biological Process; C, Cellular Component; F, Molecular Function.

^b^ GO terms with FDR<0.05 were regarded as significantly enriched GO terms.
