# Supplementary material for: Anatomy and transcript profiling of gynoecium development in female sterile Brassica napus mediated by one alien chromosome from Orychophragmus violaceus
Source: BMC Genomics. 2014 Jan 23;15:61. doi: 10.1186/1471-2164-15-61 (PMC3930543; doi:10.1186/1471-2164-15-61)
Supplement: Additional file 4: Table S3 — Significantly enriched GO terms in the DEGs specific to H3. [file 1471-2164-15-61-S4.doc]

| **GO term description** | **Ontology^a^** | **Number in input list** | **Number in BG/Ref** | **p-value** | **FDR^b^** |
| --- | --- | --- | --- | --- | --- |
| Carbohydrate metabolic process | P | 6 | 866 | 4.70E-05 | 0.00085 |
| Metabolic process | P | 17 | 10614 | 0.0039 | 0.035 |

^a^ GO term classifications: P, Biological Process; C, Cellular Component; F, Molecular Function.

^b^ GO terms with FDR<0.05 were regarded as significantly enriched GO terms.
