# Supplementary material for: Anatomy and transcript profiling of gynoecium development in female sterile Brassica napus mediated by one alien chromosome from Orychophragmus violaceus
Source: BMC Genomics. 2014 Jan 23;15:61. doi: 10.1186/1471-2164-15-61 (PMC3930543; doi:10.1186/1471-2164-15-61)
Supplement: Additional file 5: Table S4 — List of DEGs and related pathways involved in gynoecium development based on KEGG pathway and biological process of GO. [file 1471-2164-15-61-S5.doc]

| **DEGs (unigenes)** | **Gene name** | **Putative function^a^** | **Log2 Ratio(S1/H3)^b^** |
| --- | --- | --- | --- |
| **Brassinosteroid biosynthesis and** **metabolic process** | | |  |
| JCVI_38125, JCVI_42543 | *LUP2* | Lupeol synthase | -2.34, -2.10 |
| JCVI_33856, JCVI_20625 | *CAS1* | Cycloartenol synthase | -1.52, -1.27 |
| JCVI_2267 | *SMO1* | Sterol 4-alpha-methyl-oxidase | -1.03 |
| JCVI_39052 | *FACKEL* | Sterol C-14 reductase | -1.42 |
| JCVI_14504 | *SMO2* | Sterol 4-alpha-methyl-oxidase | -1.49 |
| JCVI_10359, JCVI_40245 | *DWARF5* | Δ^7^-sterol reductase | -1.36, -1.09 |
| JCVI_9676, JCVI_21095, JCVI_7150 | *DWARF1* | Calmodulin-binding | -1.53, -1.44, -1.23 |
| JCVI_27911 | *UGT73C5* | DON-Glucosyltransferase | 19.19 |
| **Adaxial/abaxial axis specification** | | |  |
| JCVI_16242, JCVI_36221 | *PHB* | [Sequence-specific DNA binding](http://www.arabidopsis.org/servlets/TairObject?type=keyword&id=22416) | -1.80, -1.40 |
| JCVI_19406, JCVI_29851 | *PHV* | [Sequence-specific DNA binding](http://www.arabidopsis.org/servlets/TairObject?type=keyword&id=22416) | -1.91, -1.44 |
| JCVI_25718, JCVI_10400 | *CNA* | [Sequence-specific DNA binding](http://www.arabidopsis.org/servlets/TairObject?type=keyword&id=22416) | -1.88, -1.18 |
| JCVI_32385 | *REV* | [Sequence-specific DNA binding](http://www.arabidopsis.org/servlets/TairObject?type=keyword&id=22416) | -1.18 |
| **Auxin transport and signaling pathway** | |  |  |
| JCVI_31884 | *PIN1* | [Transporter activity](http://www.arabidopsis.org/servlets/TairObject?type=keyword&id=4504) | -1.30 |
| JCVI_7893, JCVI_6698, JCVI_31660 | *PIN3* | [Auxin:hydrogen symporter activity](http://www.arabidopsis.org/servlets/TairObject?type=keyword&id=1628) | -2.47, -1.82, -2.66 |
| JCVI_15407, JCVI_35748 | *ABCB19* | [ATP binding](http://www.arabidopsis.org/servlets/TairObject?type=keyword&id=894), [ATPase activity](http://www.arabidopsis.org/servlets/TairObject?type=keyword&id=907) | -1.05,-1.09 |
| JCVI_32855 | *LAX3* | [Transporter activity](http://www.arabidopsis.org/servlets/TairObject?type=keyword&id=4504) | -2.19 |
| JCVI_34187 | *PID* | Serine/threonine protein kinase | -1.66 |
| JCVI_14923 | *PP2A* | Serine/threonine protein phosphatase | -1.04 |
| **Others** |  |  |  |
| JCVI_18221, JCVI_22894 | *SLG* | — | -16.34, -16.11 |
| JCVI_13562 | *SHP2* | [Protein binding](http://www.arabidopsis.org/servlets/TairObject?type=keyword&id=3877) | -1.56 |
| JCVI_35218 | *HEC1* | [Sequence-specific DNA binding transcription factor activity](http://www.arabidopsis.org/servlets/TairObject?type=keyword&id=4449) | -3.82 |
| JCVI_28865 | *SPT* | [DNA binding](http://www.arabidopsis.org/servlets/TairObject?type=keyword&id=961) | -1.25 |
| JCVI_29726 | *SRS5* | — | -2.62 |
| JCVI_9912, JCVI_18521 | *TAA1* | [Pyridoxal phosphate binding](http://www.arabidopsis.org/servlets/TairObject?type=keyword&id=8613) | -1.35, -1.63 |
| JCVI_5595, JCVI_25612 | *VDD* | [DNA binding](http://www.arabidopsis.org/servlets/TairObject?type=keyword&id=961) | -3.40, -1.16 |
| JCVI_17792 | *AGO5* | [Nucleic acid binding](http://www.arabidopsis.org/servlets/TairObject?type=keyword&id=3376) | 1,42 |
| JCVI_23062 | *PDIL2-1* | Protein disulfide isomerase | -1.01 |
| JCVI_18581 | *SEP2* | [Chlorophyll binding](http://www.arabidopsis.org/servlets/TairObject?type=keyword&id=1912) | -1.35 |
| JCVI_17089 | *PI* | [DNA binding](http://www.arabidopsis.org/servlets/TairObject?type=keyword&id=961) | 1.26 |
| JCVI_7877 | *AP3* | [DNA binding](http://www.arabidopsis.org/servlets/TairObject?type=keyword&id=961) | -1.68 |
| JCVI_13115 | *EDA14* | [Protein binding](http://www.arabidopsis.org/servlets/TairObject?type=keyword&id=3877) | 1.89 |
| JCVI_16034 | *EDA17* | [Flavin adenine dinucleotide binding](http://www.arabidopsis.org/servlets/TairObject?type=keyword&id=17564) | -1.51 |

^a^ The information is from TAIR (http://www.arabidopsis.org/index.jsp).

^b^ Negative numbers represent down-regulation and positive represent up-regulation.
